# Comparing arm to whole-body motor control disambiguates age-related deterioration from compensation

**DOI:** 10.3389/fragi.2026.1715723

**Published:** 2026-03-18

**Authors:** Robin Mathieu, Florian Chambellant, Denis Barbusse, Elizabeth Thomas, Charalambos Papaxanthis, Pauline M. Hilt, Patrick Manckoundia, France Mourey, Jeremie Gaveau

**Affiliations:** 1 Université Bourgogne Europe, INSERM, CAPS UMR 1093, Dijon, France; 2 Université Bourgogne Europe, CHU Dijon Bourgogne, INSERM, CAPS UMR 1093, Dijon, France; 3 Université Bourgogne Europe, CHU Dijon Bourgogne, Service de Médecine Interne Gériatrie, INSERM, CAPS UMR 1093, Dijon, France

**Keywords:** aging, electromyography, equilibrium, gravity, motor control, sensorimotor compensation, energetics, oxygen consumption

## Abstract

**Background:**

As the global population ages, it is crucial to understand sensorimotor compensation mechanisms. These mechanisms are thought to enable older adults to remain in good physical health, but despite important research efforts, their precise nature remains elusive and has not been definitively demonstrated. A major problem with their identification is the ambiguous interpretation of age-related alterations. Whether a change reflects deterioration or compensation is difficult to determine.

**Methods:**

To address this challenge, we examined movement efficiency in younger and older adults using two complementary approaches. In Experiment 1 (Younger, n = 20; mean age = 23.6 years, and older adults, n = 24; mean age = 72 years), we quantified energetic efficiency through the negativity of phasic EMG activity—an established marker of how the nervous system exploits gravity to minimize muscular effort—during both single-joint arm movements and whole-body actions (sit-to-stand/back-to-sit and whole-body reaching). In Experiment 2 (younger adults, n = 20; mean age = 22.9 years; older adults, n = 20; mean age = 70.6 years), we directly measured energetic cost using exhaled-gas analysis during treadmill walking under varying balance constraints.

**Results:**

In Experiment 1, older adults preserved efficient planning during arm movements, but they showed reduced gravity-related efficiency during whole-body tasks. Complementary center-of-mass analyses and optimal control simulations indicated that this reduced efficiency aligned with movement strategies favoring stability over energy minimization. In Experiment 2, older adults exhibited a disproportionately larger increase in metabolic cost and perceived effort when equilibrium demands were elevated, despite performing the same tasks as younger adults.

**Discussion:**

This supports a causal role of equilibrium constraints in decreasing walking efficiency in older adults. Overall, these results suggest that reduced movement efficiency in healthy older adults does not reflect a deterioration but rather a compensation process that adapts movement strategy to the task specificities. When balance is at stake, healthy older adults prefer stability to energy efficiency.

## 1 Introduction

Living old and healthy, also known as successful aging, is a blessing, associated nonetheless with deterioration in various organs and functions. In terms of neuromotor deterioration, aging is related to loss of muscle mass (Larsson et al., 2019), sensory receptor degradation (Goble et al., 2009; Zalewski, 2015; Saftari and Kwon, 2018), and cortical atrophy (Salat, 2004; Hoffstaedter et al., 2015). Functionally, this translates into a decline in muscle strength and power (Pousson et al., 2001; Larsson et al., 2019) and movements that tend to become slower and more variable (Darling et al., 1989; Buckles, 1993), but also into impaired balance control, characterized by less stable postural coordination (Speers et al., 2002; Anson and Jeka, 2016). If the deteriorations are too important, they lead to reductions in quality of life and, ultimately, to dependency. Although aging is associated with declines in neuromuscular and sensory function, many older adults are able to maintain functional performance through adaptive strategies. These are called compensatory strategies and, albeit often hypothesized, empirical evidence clearly demonstrating their existence or nature is still lacking. For example, they are often discussed in terms of improved stability or safety, which are critical for quality of life. However, the energetic consequences of such adaptations remain poorly understood. In particular, it is unclear whether improvements in equilibrium control necessarily come at the expense of reduced efficiency. This lack of understanding is problematic as successful aging is thought to heavily depend on compensatory processes that offset deteriorations (Baltes and Baltes, 1990; Martin et al., 2015; Zhang and Radhakrishnan, 2018). Even the most elementary concept of health includes compensatory processes at its core. The World Health Organization defined health as “a state of complete physical, mental, and social wellbeing and not merely the absence of disease or infirmity” (World Health Organization, 2002). Scientists and clinicians later redefined it even more generally as “the ability to adapt and to self-manage” (The Lancet, 2009; Huber et al., 2011). So, despite the normal deterioration associated with age, compensatory processes enable older adults to adapt and remain in good health (i.e., successful aging) and thus continue to live comfortably.

In a world with a rapidly aging population (Rudnicka et al., 2020), it is essential to understand the compensatory processes that enable older adults to remain healthy. This represents a critical step toward implementing interventions aimed at detecting, preventing, or reducing frailty and later dependency (for reviews, see Ouwehand et al., 2007; Barulli and Stern, 2013; Zhang and Radhakrishnan, 2018; Poirier et al., 2021). Compensation has long been theorized and can be defined as “a response to loss in means (resources) used to maintain success or desired levels of functioning (outcomes)” (Baltes, 1997). In the context of severe deterioration, the most basic form of compensation is the use of external aids (e.g., a crutch for walking). Such compensations are observed in frail or dependent older adults. When considering more subtle deterioration levels, identifying compensation becomes challenging. In these cases, compensatory processes enable older adults to maintain behavioral performances similar to those of younger adults, at least for the less demanding tasks of daily life (Barulli and Stern, 2013). These compensatory processes are the result of neurophysiological and behavioral adaptations that are more difficult to observe with the naked eye. Almost 30 years ago, in his famous theory of selection, optimization, and compensation, Paul Baltes and his colleagues already noted this difficulty (Baltes and Baltes, 1990; Baltes, 1997).

Since then, numerous studies have investigated compensatory processes using advanced neuroimaging and analytical approaches, showing that age-related neural alterations are often accompanied by increased recruitment of additional cortical and subcortical networks during motor and cognitive tasks (for recent reviews, see Fettrow et al., 2021; Poirier et al., 2021; Bunzeck et al., 2024). These findings suggest that compensatory neural mechanisms may help maintain functional performance despite declining efficiency. Nevertheless, behavioral compensatory processes and their subtending neural mechanisms remain largely elusive. Although they are frequently hypothesized, empirical evidence clearly demonstrating their existence or nature is still lacking. Building on the theoretical work of Krakauer et al. (2017), we recently proposed that an important reason for this failure may be that studies focusing on age-related neural alterations have used overly rudimentary behavioral paradigms (Poirier et al., 2021). Such studies have typically relied on broad behavioral metrics—muscle strength, reaction time, or movement duration—which, while capturing important motor functions, conflate multiple strategies and neural processes. Because these strategies likely degrade at different rates with age, such coarse measures risk masking compensatory adaptations by blending them with deteriorations (Poirier et al., 2021). Identifying neural compensation thus requires a more granular approach: one that links brain activity to well-characterized behavioral components. This, in turn, demands precise behavioral measurements and paradigms capable of isolating the elementary processes that shape behavior (Krakauer et al., 2017; Pereira et al., 2020; Urai et al., 2022). As such, a detailed understanding of age-related compensation at the behavioral level is a critical foundation for advancing the field.

Behavioral differences between younger and older adults may reflect compensatory responses to age-related sensorimotor decline. Particularly compelling are recent investigations that have shifted focus from broad motor performance metrics to the underlying control processes. For instance, several studies have indirectly suggested that older adults tend to favor feedforward over feedback control (Moran et al., 2014; Wolpe et al., 2016), potentially as a strategy to compensate for increased sensory noise associated with aging (Moran et al., 2014; Parthasharathy et al., 2022; Saenen et al., 2023). Other work has suggested that older adults prioritize movement efficiency over precision (Poirier et al., 2020; Healy et al., 2023), possibly to counteract elevated energetic demands in late adulthood (Didier et al., 1993; John et al., 2009; Hortobagyi et al., 2011). However, because these studies did not explicitly target compensatory mechanisms, their experimental designs were not optimized to test compensation hypotheses directly. Along these lines, it has been proposed that examining muscle recruitment strategies provides key insight into age-related differences in balance control, as differences in neuromuscular coordination distinguish older adults who recover from slip perturbations from those who fall (Sawers et al., 2017). The present study aimed to address this gap by specifically investigating task-dependent compensatory strategies in older adults.

To address this need, we conducted two experiments. The first one draw upon two distinct bodies of literature that present contrasting perspectives on motor efficiency in aging. On one hand, a series of studies has shown that the brain plans efficient arm movements by leveraging the mechanical effects of gravity to reduce muscular effort (Berret et al., 2008; Crevecoeur et al., 2009; Gaveau and Papaxanthis, 2011; Gaveau et al., 2014; 2016; 2021; Gueugneau et al., 2023; for a review, see White et al., 2020). Across a wide range of arm movement tasks—including single- and multi-degree-of-freedom pointing, drawing, reach-to-grasp actions, and object transport—previous studies consistently support the idea that arm motor patterns are shaped by an optimization principle: movements are organized to exploit gravity in order to minimize energy expenditure (Papaxanthis et al., 1998; Papaxanthis et al., 2005; Le Seac’h and McIntyre, 2007; Berret et al., 2008; Paizis et al., 2008; Crevecoeur et al., 2009; Gaveau et al., 2011; Gaveau and Papaxanthis, 2011; Yamamoto and Kushiro, 2014). Notably, recent findings suggest that this capability is not only preserved but may even be enhanced in older adults (Huang and Ahmed, 2014; Poirier et al., 2020; Healy et al., 2023; Summerside et al., 2024). To ensure that our protocol could be completed within a single session per participant, we selected a single representative arm task that captures this optimization principle. On the other hand, research on whole-body movements paints a markedly different picture. Studies investigating movements that engage the entire body report that older adults tend to plan less efficient trajectories (Paizis et al., 2008; Schrack et al., 2012; Casteran et al., 2018, Payne et al., 2021). This inefficiency is especially surprising given that such movements demand more energy in older adults than in younger ones (Hortobagyi et al., 2003; 2011; Julius et al., 2012; VanSwearingen and Studenski, 2014). Given that older adults retain the ability to plan efficient movements—demonstrated by findings from the arm movement literature—one might speculate that the decreased efficiency observed in whole-body movements reflects a form of age-related compensation, specifically a strategic adaptation in motor planning. However, this interpretation remains speculative due to the differing experimental paradigms and measurement techniques employed across these two lines of research. More critically, an alternative explanation could be supported by numerous other studies: the reduced efficiency in whole-body movements may instead result from a deterioration in the ability to generate efficient motor patterns (Goodpaster et al., 2006; Vernazza-Martin et al., 2008; Quinlan et al., 2018; Henry and Baudry, 2019). To adjudicate between these competing interpretations, we investigated both arm and whole-body movements using identical methodologies. Specifically, we tested the hypothesis that age-related declines in whole-body movement efficiency reflect a compensatory strategy—that is, an adaptive shift in motor planning to accommodate other deteriorating sensorimotor functions.

Building on this first set of results, we conducted a second experiment designed to more directly quantify energetic efficiency and to test for a causal role of equilibrium constraint in decreasing efficiency in older adults. Previous work has shown that whole-body movements become increasingly costly with age (Didier et al., 1993; Hortobagyi et al., 2003; 2011; John et al., 2009), and that older adults would adopt movement strategies that prioritize equilibrium maintenance over energetic minimization (Paizis et al., 2008; Casteran et al., 2018). However, these conclusions have largely been based on kinematic proxies or indirect estimates of effort. To more precisely assess whether heightened equilibrium constraints disproportionately reduce energetic efficiency in older adults, we relied on exhaled-gas analysis—a gold-standard, metabolic measurement technique that has been widely used to investigate walking efficiency (Collins et al., 2018; Donelan et al., 2002; VanSwearingen et al., 2009). This second experiment therefore tested the prediction, grounded in prior work, that increasing balance demands would entail a greater energetic increase in older adults than in younger adults.

Overall, Experiment 1 assesses the association between decreased walking efficiency and improved equilibrium control in older adults, while Experiment 2 evaluates the causal role of equilibrium constraints in decreasing walking efficiency in older adults.

## 2 Methods

### 2.1 Participants

Because we had no prior data to calculate the ideal sample size, we included as many participants as possible over a fixed recruitment period. Twenty younger adults (23.6 ± 2.1 y.o.) and twenty-four older adults (72 ± 5.3 y.o.) were included in the first study. Twenty younger adults (22.9 ± 2.8 y.o.) and twenty older adults (70.6 ± 4.6 y.o.) were included in the second experiment. Among them, three younger and eight older adults took part in both experiments. All participants gave their oral informed consent. Participants had normal or corrected-to-normal vision and did not present any neurological or musculo-articular disorders. The laterality index of each participant of the first experiment was superior to 60 (Edinburgh Handedness Inventory, (Oldfield, 1971), indicating that all participants were right-handed. The study was carried out following legal requirements and international norms (Declaration of Helsinki, (World Medical Association, 1964), and approved by the French National Ethics Committee (2019-A01558-49). Each participant was included in the study by a medical doctor.

### 2.2 Experiment 1

#### 2.2.1 Experimental protocol

**FIGURE 1:**
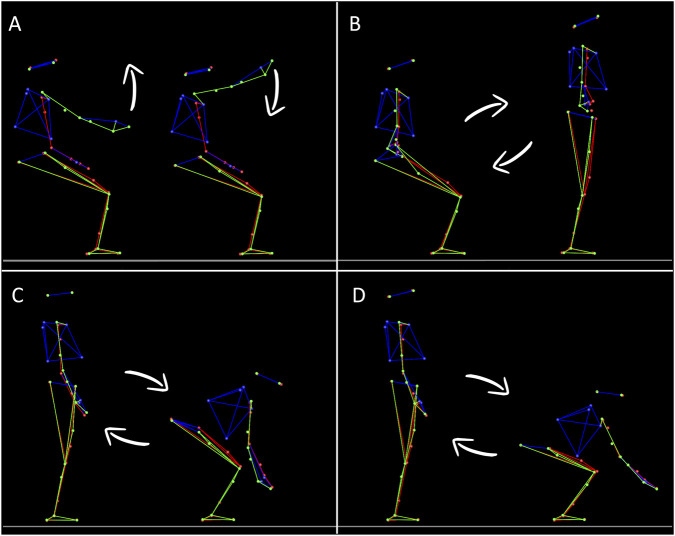
Illustration of the four tasks. Each panel shows the extreme body positions between which participants performed their movements. Each position was alternatively the starting or ending target of a movement, depending on movement direction. **(A)** Single degree of freedom arm movements flexion/extension around the shoulder joint (flexion/extension). Participants performed upward and downward arm movements. **(B)** Sit-to-stand/Back-to-sit movements. Participants performed vertical multi-articular whole-body movements to either stand up from the stool (upward movement) or sit on it (downward movement). **(C)** Whole-body reaching task toward a near target (15% of the participant height). Participants performed vertical multi-articular whole-body movements to either reach towards targets that were located nearby the floor (downward movement) or to bounce back from this position toward a resting vertical standing position (upward movement). **(D)** same as C but with targets that were placed farther away on the antero-posterior axis (30% of the participant height).

All participants performed four tasks in a randomized order. These tasks either required moving the arm only (Figure 1A) or the whole-body (Figures 1B–D). Whole-body movements consisted of sit-to-stand/back-to-sit (STS/BTS, Figure 1B), whole-body reaching toward near targets (WBR D1, Figure 1C), and whole-body reaching toward distant targets (WBR D2, Figure 1D). The arm task was selected because it is the reference task that has been studied to demonstrate how muscle patterns take advantage of gravity effects to save energy (Gaveau et al., 2016; 2021). The whole-body tasks were selected because they include an equilibrium constraint, represents movements of daily life, and they have been investigated in previous studies (Millington et al., 1992; Mourey et al., 1998; Manckoundia et al., 2006; Paizis et al., 2008; Casteran et al., 2018; Jeon et al., 2021).

##### 2.2.1.1 Trial organization

The organization of a trial was similar for all tasks. It was carried out as follows: i) the experimenter indicated to get ready; ii) the participant adopted the requested initial position; iii) after a brief delay (∼1 s), the experimenter verbally informed the participant that she/he was free to reach the requested final position whenever she or he wanted (i.e., reaction time was not constrained); iv) the participant was requested to maintain the final position for a brief period (about 1 s); v) the experimenter instructed the participant to move back to the starting position (reversed movement) whenever desired; vi) lastly, the participant was asked to relax. A short rest period (∼20 s) separated trials to prevent muscle fatigue. Additionally, participants were free to rest as long as they wanted between blocks. Participants were allowed to perform a few practice trials (∼3 trials) before each block. Low-speed and high-speed blocks were similar except that the instructions were to perform the movements in roughly 5 s or as fast as possible, respectively.

##### 2.2.1.2 ARM task

This task was similar to those used in prior studies investigating human movement adaptation to gravity (Le Seac’h & McIntyre, 2007; Gentili et al., 2007; Gaveau and Papaxanthis, 2011; Gaveau et al., 2014; Yamamoto and Kushiro, 2014; Hondzinski et al., 2016; Gaveau et al., 2016; Poirier et al., 2020; Gaveau et al., 2021; Poirier et al., 2022). Participants used their right arm to perform vertical, single-degree-of-freedom flexion and extension movements around the shoulder joint. Two blocks of arm movements were executed in randomized order: one block included twelve slow movements (six upward, six downward), and the other consisted of 24 fast movements (twelve upward, twelve downward). Two circular targets (3 cm in diameter) were positioned in front of the participant’s right shoulder, within a parasagittal plane, at a distance equal to the length of their fully extended arm plus 2 cm. The required movement amplitude between the two targets was 45°, corresponding to shoulder elevation angles of 112.5° (upward target, 22.5° above horizontal) and 67.5° (downward target, 22.5° below horizontal).

##### 2.2.1.3 STS/BTS task

This task was similar to those of previous studies (Millington et al., 1992; Mourey et al., 1998; Manckoundia et al., 2006; Jeon et al., 2021). Participants were seated on an armless stool whose height was adjusted to correspond to 30% of the participant’s height. The hands were positioned on the hips, and the participants were instructed to maintain their back in a vertical orientation. Participants were asked to stand up from the stool, make a short pause (about 2s), and then sit back on the stool. Similarly to arm movements, participants executed two blocks of movements in a randomized order. One block consisted of six slow movements, and the other consisted of 12 fast movements.

##### 2.2.1.4 WBR task

This task was similar to those of (Paizis et al., 2008; Casteran et al., 2018). Starting from an upright position, we asked participants to perform whole-body reaching movements (WBR) toward two targets nearby the floor with their two index fingers (10% of their heights above the floor). The two targets (4 × 2 cm) were spaced by 0.5 m on a medio-lateral axis and centered on the participant’s sagittal axis. They were placed in front of the participant at two different distances, corresponding to 15% (D1) or 30% (D2) of their height on the antero-posterior axis. Distances were measured from the participant’s big toe. Similarly to the previous two tasks, for each distance and in a randomized order, participants executed two blocks of trials performed at two different speeds. One block consisted of six slow movements and the other one of twelve fast movements (total of four blocks: two speeds × two distances).

#### 2.2.2 Data collection

##### 2.2.2.1 Kinematics

We recorded the position of all markers with an optoelectronic motion capture system (Vicon system, Oxford Metrics, United Kingdom; 18 cameras) at a sampling frequency of 200 Hz. The spatial variable error of the system was less than 0.5 mm. We used the standard plug-in Gait full body model (Vicon, Oxford Metrics, United Kingdom) using 39 reflective markers on the participant’s head (temples and backs of the head to form a rigid plan with the head), back (C7, T10 and on the right scapula), torso (jugular notch where the clavicles meet the sternum and on the xiphoid of the sternum), shoulders (acromion), arms (upper lateral 1/3 for the left arm, and 2/3 for the right arm), elbows (lateral epicondyle), forearms (lower lateral 1/3 for the left forearm, and 2/3 for the right forearm), wrists (both cubitus styloid processes), hands (middle of the proximal knuckle of the index), pelvis (anterior and posterior superior iliac spine), thighs (upper lateral 1/3 for the left leg, and 2/3 for the right leg), knees (lateral side of the flexion-extension axis), calves (upper lateral 1/3 for the left calf, and 2/3 for the right calf), ankles (lateral malleolus), and feet (second metatarsal head and heel). The markers on the scapula, on the arms, on the forearms, on the thighs, and on the calves have been deliberately placed asymmetrically so that the model can best dissociate the right and left sides; these markers are not used for the analyses presented in this manuscript.

##### 2.2.2.2 EMG

We placed sixteen bipolar surface electrodes (Cosmed, wireless pico EMG, sampling frequency: 1000 Hz) on the anterior (AD) and posterior (PD) heads of the deltoid, vastus lateralis (VL), biceps femoris (BF), spinal erectors on L1 (ESL1) and on T7 (EST7), the soleus (SOL), and on the tibialis anterior (TA) to record EMG activity. Electrodes were placed bilaterally. The location of each electrode was determined following the recommendations from Barbero et al. (2012). The Giganet unit (Vicon, Oxford Metrics, United Kingdom) synchronously recorded kinematic and EMG data.

#### 2.2.3 Data analysis

We processed kinematic and EMG data using custom programs written in Matlab (Mathworks, Natick, MA, 2021). Data processing was inspired by previous studies (Gaveau et al., 2021; Poirier et al., 2022) and was similar for all tasks.

##### 2.2.3.1 Kinematics analysis

First, we filtered position using a third-order low-pass Butterworth filter (5 Hz cut-off, zerophase distortion, “butter” and “filtfilt” functions). We then computed the amplitude of the movement using steady phases (200 ms for fast movements and 500 ms for slow movements) before and after the movement, using the marker of the right shoulder (for whole-body movements, see Supplementary Figure S1) or the right finger (for arm movements). The amplitude was computed on the Z-axis for fast movements and on the X, Y, and Z-axes for slow movements. For slow movements, we used 3D position to minimize detection error on signals that were more variable than those obtained during fast movements. Last, we automatically defined movement onset and offset as the moments when the displacement rose above or fell below a threshold corresponding to 5% and 95% of the total movement amplitude, respectively.

On behalf of using the kinematics to define the start and end of movement, we analyzed the displacement of the Center of Mass (CoM) in three dimensions to understand how equilibrium was maintained during the whole-body tasks. This was done to replicate the work of Paizis et al. (2008) and Casteran et al. (2018) and, more importantly, to perform a straightforward analysis to test whether our primary criterion, measured via electromyographic activity, is linked to a simple and interpretable equilibrium control modification. The CoM was calculated kinematically using Vicon motion capture. Our analysis utilized a seven-segment mathematical model incorporating rigid segments: Trunk, Thigh, Shank, Foot, Upper Arm, Forearm, and Hand. This model was implemented following the methodologies previously described by Stapley et al. (1999) and Berret et al. (2008), in which whole-body kinematics are reconstructed from linked rigid segments. Segment anthropometric parameters (segment mass as a percentage of total body mass, segment length as a percentage of total height, and the relative position of each segment’s center of mass) were obtained from Winter (2009), as done in similar studies (Stapley et al., 1999; Berret et al., 2008). We determined movement onset and offset using velocity profiles. We used a threshold of 5% of the peak velocity. We further explored the kinematics of the whole-body tasks using two simple parameters: i) the total displacement of the CoM, calculated as the distance between the start and end positions and normalized by the participant’s height; and ii) the amplitude of the CoM peak velocity. We focused on downward movements, as these are the ones that have been studied and present the greatest challenge to balance. The specific process to compute criteria used by previous studies (Paizis et al., 2008; Casteran et al., 2018) is detailed and available in Supplementary Figure S2.

##### 2.2.3.2 EMG analysis

Below, we describe the procedures used to obtain EMG measures, following methodologies established in previous studies.

###### 2.2.3.2.1 Pre-processing

EMG signals were first rectified and filtered using a bandpass third-order Butterworth filter (bandpass 30–300 Hz, zero-phase distortion, “butter” and “filtfilt” functions) followed by a low-pass third-order Butterworth filter (low-pass frequency: 5 Hz) to highlight important features of muscular activities. Signals were integrated using a 100 ms sliding window using trapezoidal numerical integration from Matlab (Mathworks, 2021) using the built-in “trapz” function. For fast movements, EMG signals were cut off from 240 ms before movement onset to 90 ms after movement offset. For slow movements, EMG signals were cut off from 75 ms before movement onset to 75 ms after movement offset. These timing values were obtained from preliminary analyses detecting EMG activity start and stop before and after all movements. The result is the average of all participants. Importantly, those values were kept constant for all participants and, thus, should not bias group comparisons.

###### 2.2.3.2.2 Phasic/tonic separation

We then computed the phasic component of each EMG signal using a well-known subtraction procedure that has mostly been used to study arm movements (Flanders and Herrmann, 1992; Buneo et al., 1994; Flanders et al., 1994; D’Avella et al., 2006; D’Avella et al., 2008; Gaveau et al., 2021). This processing allows quantifying how much the central nervous system takes advantage of the gravity torque when moving the body in the gravity environment (Gaveau et al., 2021; Poirier et al., 2022, Poirier et al., 2023b). Here, we adapted this procedure to study whole-body movements, which involve greater complexity than single-degree-of-freedom movements. First, the tonic signal was obtained from the six slow movements (average of the six trials). For that purpose, the cut movements (as described earlier) were normalized in duration to be finally averaged together in one tonic signal. Second, to improve signal to noise ratio, EMG traces of fast movements were ordered according to movement mean velocity and averaged across two trials (from the two slowest to the two fastest movements). This resulted in six EMG traces to be analyzed for each block. Each set of two traces was normalized in duration (corresponding to the mean duration of the two traces) before averaging. Third, the phasic component was obtained by subtracting the tonic EMG from the EMG trace of each pair of fast movements. Finally, to set the data of all participants on a common scale, phasic activity was normalized by the maximal EMG value recorded in each task for each participant.

###### 2.2.3.2.3 Quantifying negativity

**FIGURE 2:**
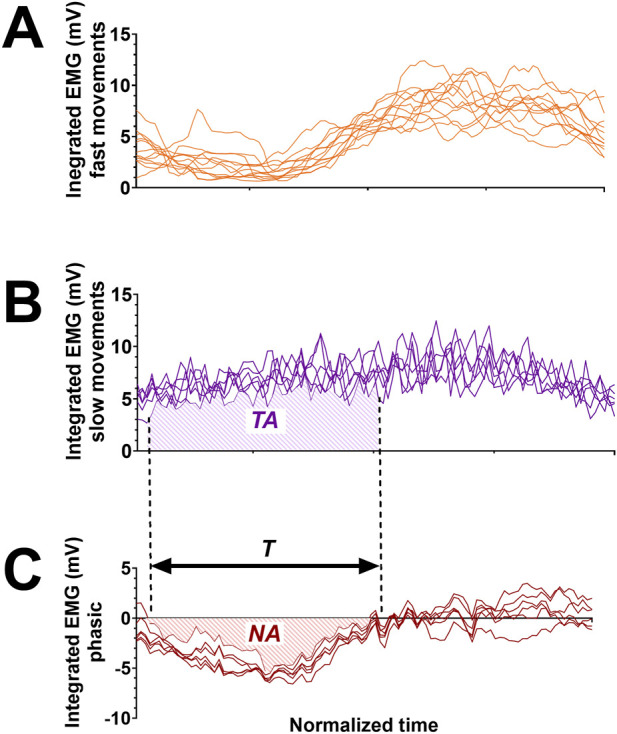
Illustration of the calculation method to obtain phasic EMG components. Electromyographic signals (mV) are presented as a function of time. Pattern duration and amplitude are normalized (see methods). **(A)** Six integrated Vastus Lateralis EMG signals during fast BTS movements of a typical participant (BTS: Back-to-sit); **(B)** Six integrated Vastus Lateralis EMG signals recorded during slow BTS movements of a typical participant. These signals represent the tonic component. TA: Tonic Area integrated on the tonic signal between the negativity onset and offset; **(C)** Integrated phasic EMG component computed using the six fast **(A)** and slow movements **(B)**. The phasic is calculated by subtracting the mean of the slow acquisitions from the fast acquisitions (Phasic = Fast–Tonic). T: the duration of the negative epoch normalized by movement duration and NA: the Negative Area integrated on the phasic signal between negativity onset and offset.

We defined negative epochs as intervals where the phasic EMG signal was inferior to zero minus three times the standard deviation of the stable phase preceding the movement, and this for at least 40 ms. This duration has been chosen after preliminary tests to avoid detecting false-positives. We kept it constant for all analyses. We used this value as a threshold to automatically detect negativity onset and offset. On each negativity phase, we computed: i) a negativity index, as defined in Equation 1, with NA the Negative Area integrated on the phasic signal between negativity onset and offset, TA the Tonic Area integrated on the tonic signal between the negativity onset and offset, and T the duration of the negative epoch normalized by movement duration (see Figure 2). This value is always negative or null. The lower the value, the greater the efficiency; ii) negativity occurrence, defined as the number of trials where a negative epoch was automatically detected, divided by the total number of trials in the condition; iii) negativity duration, defined as the duration of the negative epoch normalized by movement duration; iv) negativity amplitude, defined in Equation 2, during the negative period. A value of −100 indicates that the muscle was completely relaxed and a value of 0 indicates that the muscle exactly compensated the gravity torque.

Negativity Index=T×NA / TA (1)

Negativity amplitude=Phasic value/Tonic Value×100 (2)

###### 2.2.3.2.4 Muscles selection

It was recently shown that the phasic EMG activity of antigravity muscles, those that pull against the gravity vector, consistently exhibits negative epochs (Gaveau et al., 2021; Poirier et al., 2022; Thomas et al., 2023; Chambellant et al., 2024) when the arm acceleration sign is coherent with the gravity acceleration sign (i.e., in the acceleration phase of downward movement and in the deceleration phase of upward movements). This observation likely reflects an optimal motor strategy where muscle activity is decreased when gravity assists arm movements, thereby saving energy (Gaveau et al., 2021). In the present study, the antigravity muscles are: i) the Anterior Deltoïd (DA), flexing the shoulder joint; ii) the Vastus Lateralis (VL), extending the knee joint; iii) the Erector Spinae L1 (ESL1), extending the rachis; iv) the Erector Spinae T7 (EST7), extending the rachis; v) the Soleus (SOL), flexing the ankle in the plantar direction. Because the Erector Spinae T7 and the Soleus muscles did not play a strong focal role but a rather postural one in the present tasks, we focused our analyses on the remaining three muscles (DA, VL, and ESL1). Probing the activation of a postural muscle, per definition, is not appropriate to test whether the nervous system takes advantage of gravity effects to move. Compared to other joints (e.g., hips and knees), the ankle and upper rachis were only minimally mobilized in the tasks we investigated here (see stick diagrams in Figure 1). Including these muscles in our analyses would thus add noise to our dependent variables and likely impede our ability to test our hypothesis. Therefore, we focused on DA during arm movements and on VL and ESL1 during movement of the entire body.

As is often the case with EMG recordings, some of the EMG signals exhibited aberrant values. Those signals are usually due to poor contact between the electrodes and the skin. Supplementary Table S1 summarizes the issues encountered with all electrodes and participants.

#### 2.2.4 Machine learning

Machine learning was used to verify that the muscles selected for our analysis truly conveyed age-related differences in whole-body movement control. Specifically, successful discrimination between younger and older adults based on antigravity muscle activation patterns demonstrated that these muscles contained relevant information. Similar applications of machine learning to EMG-based discrimination have been reported in Chambellant et al. (2024), Thomas et al. (2023), and Tolambiya et al. (2011).

We used custom Matlab scripts to perform all machine learning analyses. The left ESL1 was not considered for these analyses because the electrode was defective for several younger participants (see Supplementary Table S1). This control analysis aimed to identify muscle activation patterns that carried significant information about age-related differences in movement control. We report Linear Discriminant Analysis results for clarity and interpretability, and verified that similar conclusions were obtained using Quadratic Discriminant Analysis and Support Vector Machine, thereby ensuring robustness of the conclusion.

The input data was the phasic EMG signals of the 15 muscles taken individually or the whole set at once. These vectors were fed to the algorithms using binary classification setups, where the algorithm learned to distinguish between the EMGs of the two groups. To ensure robustness of the results, we employed a five-fold cross-validation method. This involved splitting the whole dataset into five sets while ensuring equal representation of both directions in each set. The algorithm was trained on four of those sets before being evaluated on the fifth set (containing data unknown to the trained algorithm). This operation was repeated five times, so each set was tested once. Cross-validation allowed computing the average accuracy and its variance across the testing sets, thereby providing a reliable estimate of the accuracy obtained by the algorithm. Finally, we could compare the accuracy of the algorithm for each muscle.

#### 2.2.5 Optimal control simulations

Here we were interested in verifying mathematically whether our results support the hypothesis of an age-related change in strategies. One way of testing changes in motivations that define shifts in strategy is to use optimal control algorithms. We focused here on the whole-body reaching task, which has already been modelled in previous studies (Casteran et al., 2018; Hilt et al., 2016). We reused the same musculoskeletal model (see detailed description in Supplementary Method 1). As in previous studies, we modeled the dynamics of the musculoskeletal system using a series of articulated rigid bodies with joints primarily moving in the sagittal plane, neglecting viscous friction and elastic tissue properties. The system dynamics were expressed using the classical Lagrangian formalism, while muscle dynamics were modeled as a first-order low-pass filter.

Our contribution goes beyond these prior works in two ways. First, instead of reproducing a single optimal solution, we systematically varied the relative weights of two cost components—movement efficiency (angular jerk and torque work) and postural stability (sum of torques)—to generate families of solutions representing different strategic preferences. Second, we extracted center-of-mass displacement and velocity from each solution in order to compare them quantitatively to experimental age-group differences, which was not done in previous models.

The model simulations were conducted using the GPOPS software (Rao et al., 2010).

#### 2.2.6 Univariate statistics

After an initial kinematic analysis (detailed in the results section), we observed a difference in movement duration between younger and older adults (conducting a repeated measure analysis of variance with a between factor *Age* with two levels: Young/Older and a within factor *Task-type* with two levels: Arm/Whole-body movements). Because movement duration is known to influence phasic EMG negativity (Poirier et al., 2023b), we added movement duration as a covariate, as our objective was to isolate age-related differences in motor control strategies rather than differences in movement speed. Before conducting the analyses of covariance, we verified in JASP (JASP Team, 2025) that the data met the necessary assumptions (e.g., normality, homogeneity of variances). Two ANCOVA analyses were carried out. The first analysis served as our primary test, designed to address the main question of whether age-related compensation mechanisms differed across task types; focusing on this single primary comparison allowed us to avoid inflating Type I error through multiple comparisons. Specifically, we used a mixed ANCOVA with a between factor *Age* (two levels: Young/Older) and a within factor *Task-type* (two levels: Arm/Whole-body movements) to test whether age effects on movement control depended on the type of task being performed. Second, to detail the age differences observed during movements of the entire body, we used a mixed ANCOVA with a between factor *Age* (two levels: Young/Older) and a within factor *Whole-Body-Tasks* (three levels: STS_BTS/WBR D1/WBR D2). Additionally, we conducted a separate mixed ANCOVA to test for a distance effect, using only the WBR D1 and WBR D2 conditions as the two levels of the within-subject factor. In all cases, the significance level was set to 0.05.

To test for a possible beneficial effect (i.e., compensation) of the EMG alterations that we observed with aging, we performed a kinematic analysis of the center of mass. We then used independent Student’s t-tests and Pearson’s correlation coefficients to study potential differences between groups and associations between variables.

### 2.3 Experiment 2

#### 2.3.1 Experimental protocol

**FIGURE 3:**
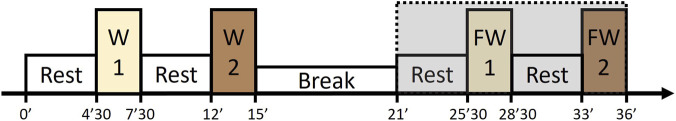
Organization of the experiment. Timeline of the experiment with two blocks of walking at 2.5 km/h (W1: normal walking, W2: walking on a line), and two blocks of walking at 4 km/h (FW1, FW2, shaded blocks, only for young participants). Each block lasted 3 min s and blocks were interspersed with a 4.5 min rests period during which the participants sat still. 6 min rest breaks were also included halfway through the experiment to remove the mask and free participants to drink and stretch their legs.

The goal of the experiment was to quantify energy consumption during a balance-related tasks. The tasks were performed using a block design, as it was done in previous studies (see Figure 3, Huang and Ahmed, 2014; Selinger et al., 2015). The aim was to reach a stabilized state of energy consumption.

##### 2.3.1.1 Walking task

**FIGURE 4:**
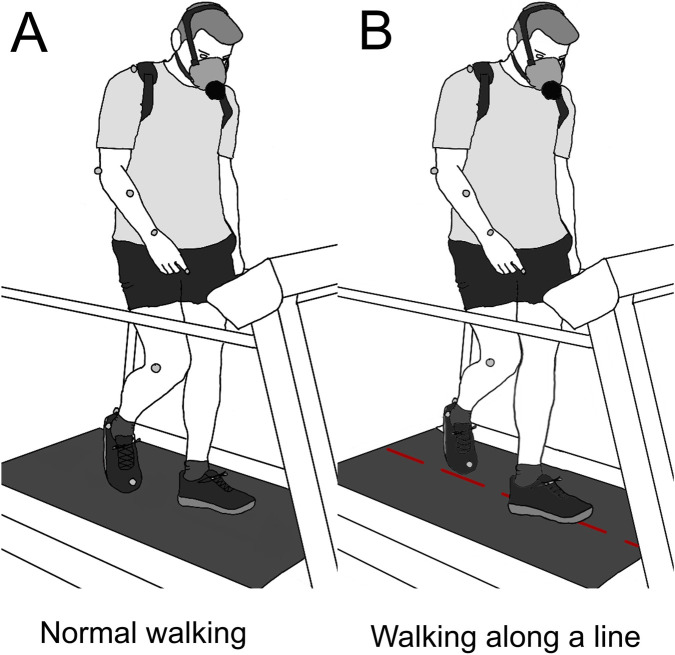
Illustration of the tasks. **(A)** Unconstrained walking condition: the participant walks at a constant speed without any additional task. **(B)** Stability-constrained walking condition: the participant is instructed to step on a narrow virtual path marked on the treadmill, thereby increasing the demand for balance control. Both conditions are performed with simultaneous recording of kinematics, and exhaled gas activity.

Participants were asked to walk on a treadmill (TecMachine ®) under two conditions: walking as usual and walking along an imaginary line (see Figures 4A,B). In the imaginary line condition, the instruction for the participant was to place their feet on this line, thereby reducing the base of support. The line was imaginary on the moving belt itself, but a physical reference line was drawn on the fixed front part of the treadmill to provide a visual cue. Older participants completed two 3-min walking blocks at 2.5 km/h. Younger participants also completed two walking blocks at 2.5 km/h, as well as two additional blocks at 4 km/h (see gray shaded block in Figure 3). The blocks were separated by 4-min and 30-s rest periods, during which participants sat peacefully on a chair. The order of the equilibrium conditions was randomized. Before the experiment began, participants were given a 5-min period to familiarize themselves with walking on the treadmill.

##### 2.3.1.2 Trial organization

The organization was carried out as follows: i) the experimenter indicated to get in place 30 s prior to the beginning of the block (to get on the treadmill), ii) the experimenter gradually increases speed of the treadmill to reach the target speed.

#### 2.3.2 Data collection

##### 2.3.2.1 Kinematics

We used a custom model (Vicon, Oxford Metrics, United Kingdom). 14 reflective markers were placed on the participant’s shoulder (acromion), arm (upper lateral ½, marker for model creation), elbow (lateral epicondyle), wrist (intern cubitus styloid process), finger (index finger nail), pelvis (anterior superior iliac spine and second sacrum vertebra), knee (lateral side of the flexion-extension axis), and feet (second metatarsal head, heel and the shoe strap, marker for model creation).

We recorded the position of all markers with an optoelectronic motion capture system (Vicon system, Oxford Metrics, United Kingdom; 18 cameras) at a sampling frequency of 200 Hz. The spatial variable error of the system was less than 0.5 mm.

##### 2.3.2.2 Exhaled gas

Energy consumption was assessed with a Cosmed K5 portable metabolic system employing breath-by-breath mode (BxB), held on the back of participants. Prior to each data collection, the metabolic system underwent calibration using certified gas mixtures and a 3L calibration syringe across various flow rates. All data were subsequently adjusted for standard temperature, humidity, and pressure by the metabolic system.

##### 2.3.2.3 Effort perception

We measured perception of effort, defined by (Marcora, 2010) as “the conscious sensation of how hard, heavy, and strenuous a physical task is”. We used the 6–20 RPE scale proposed by (Borg, 1962) with standardized oral instructions given to each participants. The perception of effort was measured right after each block and was requested for the entire block that had been completed.

#### 2.3.3 Data analysis

We processed data using custom programs written in Matlab (Mathworks, 2021).

##### 2.3.3.1 Kinematics analysis

First, we filtered position using a third-order low-pass Butterworth filter (5 Hz cut-off, zerophase distortion, “butter” and “filtfilt” functions). We computed three criteria: i) the medio-lateral distance between feet, ii) the step rate, and iii) the step length and its variability. To compute the distance between feet during the tasks, we calculated the difference along the medio-lateral axis between the average position of the left foot and that of the right foot. We performed peak detection of the right foot position to determine the number of steps, which we then divided by the time spent between the first peak and the last. Finally, we computed the step length by detecting peak on the right foot position along the antero-posterior axis and we calculated the prominence of these peaks.

##### 2.3.3.2 Energy consumption analysis

We used VO2 and VCO2 measurements and applied the Brockway equation (Brockway, 1987; Equation 3) to calculate metabolic power in watts:

$$P=C_{1}\times VO_{2}+C_{2}\times V{CO}_{2}$$ (3)

with P the power, $C_{1}$ an empirical coefficient equal to 16.38, $VO_{2}$ the volume of dioxygen consumed, $C_{2}$ an empirical coefficient equal to 4.64, $V{CO}_{2}$ the volume of dioxygen consumed (Brockway, 1987; Kipp et al., 2018). Additionally, we normalized this value by dividing it by body mass to express metabolic power in W/kg. Then, by averaging the last minute of the block, we computed the energy consumption rate. We used the average of the baseline between −1 min and −30 s before each block to subtract the steady state consumption.

#### 2.3.4 Univariate statistics

We performed repeated measure analyses of variance (ANOVA) using JASP software. We used a mixed ANOVA with a between factor *Age* (two levels: Young/Older) and a within factor *Task-type* (two levels: moderate and high equilibrium constraint) to test whether the effect of age on movement control varied with the level of equilibrium constraint.

## 3 Results and discussion

### 3.1 Experiment 1

The Machine Learning analysis revealed that antigravity muscles contained important information, allowing separating age-groups with some of the best success-rates (see Supplementary Figure S3 for results regarding LDA accuracy). The vastus lateralis (VL) and the spinal erectors on L1 (ESL1) achieved the best classification accuracies of 57.72% and 59.51% respectively (considering that these classifications are significantly better than chance if they are above 52.5% according to a fairness test). The main results presented in this article therefore originate from analyses of the muscles that show the most information to distinguish younger from older adults during whole-body movement.

**FIGURE 5:**
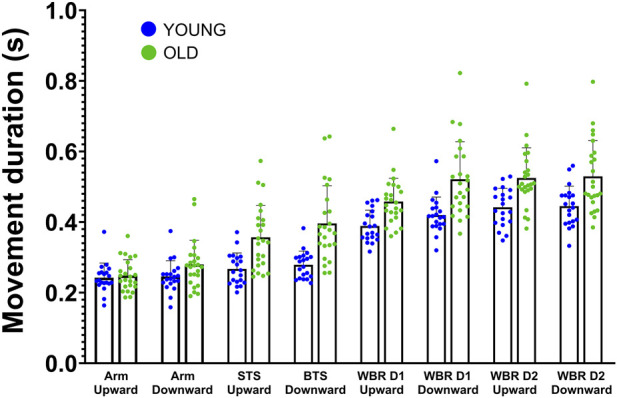
Mean ± SD movement durations (s) for fast movements performed in all tasks and both groups (STS: Sit-to-stand, BTS: Back-to-sit, WBR: Whole-body-reaching, D1: Short distance = 15% of the height of the participant, and D2: Long distance = 30% of the height of the participant). Each point corresponds to the average duration of the trials of one participant. The blue points represent the young participants, and the green points correspond to the older participants.

Movement duration of fast movements varied between tasks and was slightly reduced in younger compared to older participants (see Figure 5; Supplementary Table S2 for detailed results). Overall, older adults were 3.5% slower than younger adults. A repeated measures ANOVA revealed that this age-difference was significant ( $F_{\left(1,42\right)}$ = 14.5, P = 4.58E-05, ${ƞ}^{2}$ = 0.256). For this reason, we used movement duration as a covariate in the following statistical analyses. Nevertheless, as revealed in Figure 5, it is important to note that an important number of older adults moved with durations that were similar to those of younger adults.

**FIGURE 6:**
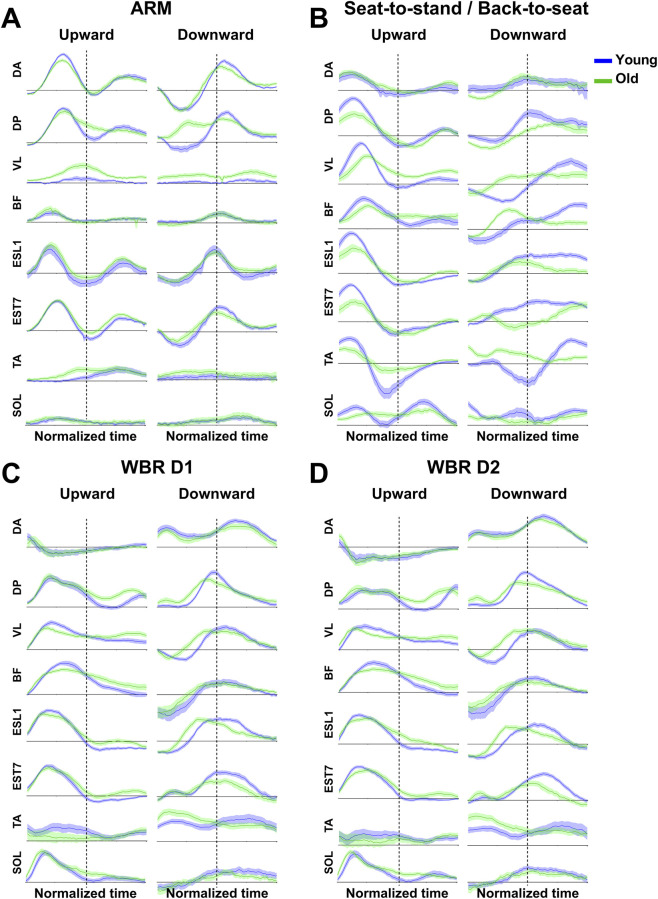
Mean (±SE) integrated phasic EMGs recorded for both groups (n = 20 for younger and n = 24 for older) during arm **(A)**, Sit-to-stand/Back-to-sit **(B)**, and whole-body reaching movements **(C)** for short distance D1 and **(D)** for long distance D2). Blue traces present EMGs recorded for younger participants, while green traces present EMGs recorded for older participants. The dotted line divides the movement into two phases: the first corresponds approximately to acceleration, and the second to deceleration. (DA: Anterior deltoid, DP: Posterior deltoid, VL: Vastus Lateralis, BF: Biceps Femoris, ESL1: Erector Spinae in L1, EST7: Erector Spinae in T7, TA: Tibialis Anterior, SOL: Soleus).

Figure 6 displays average phasic EMG profiles for each muscle, direction, and task. As recently reported, phasic EMG signals of arm movements show negative phases during the deceleration of upward and the acceleration of downward arm movements, respectively (Figure 6A), i.e., where gravity torque helps generate the arm’s motion (Gaveau et al., 2021; Poirier et al., 2022; Poirier et al., 2023a). Previous work also demonstrated that this negativity is not erratic but systematic and indicated that muscles contract less than necessary to compensate for gravity effects. It is therefore especially prominent on antigravity muscles and reveals that the central nervous system (CNS) exploits gravity effects to produce efficient movements, i.e., motor patterns that save unnecessary muscle work. Here, we extend this result to movements performed with the entire body. Indeed, for STS/BTS and WBR movements, Figures 6B–D unveils phasic EMG negativity during the deceleration of upward movements and the acceleration of downward movements, i.e., when gravity helps to produce the motion. This first qualitative result reveal that movements that are performed with the entire body, similar to more focal arm movements, exploit gravity effects to save unnecessary muscle work (Gaveau et al., 2021). Notably, the present results qualitatively reveal that older adults also use such an efficient strategy, both when moving their arm and their entire body.

#### 3.1.1 Main analysis

**FIGURE 7:**
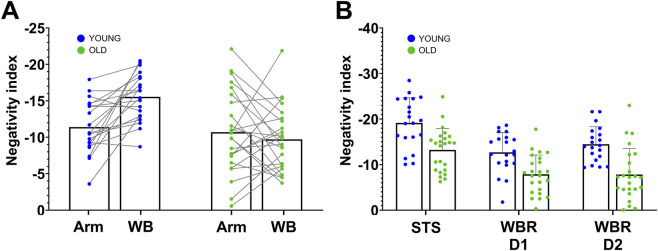
Negativity index computed for **(A)** arm and whole-body movements in both groups (WB: Whole-Body combines sit-to-stand/back-to-sit, whole-body reaching from D1 and whole-body reaching from D2) and **(B)** each whole-body task (STS/BTS: sit-to-stand/back-to-sit, WBR D1: whole-body reaching from D1 and WBR D2: whole-body reaching from D2). The negativity index, defined as T x NA/TA, with NA the Negative Area integrated on the phasic signal between negativity onset and offset, TA the Tonic Area integrated on the tonic signal between negativity onset and offset, and T the duration of the negative epoch normalized by movement duration. The blue points correspond to the younger participants, and the green points correspond to the older participants. Each point corresponds to the mean value of one participant (mean across trials and antigravity muscles, and/or tasks).

Following our primary hypothesis, we first analyzed a single metric that quantified phasic EMG negativity within an averaged muscle activation pattern. For whole-body tasks, we averaged the vastus lateralis and erector spinae (L1), while for arm tasks, we used the anterior deltoid. We verified that these muscles contained information relevant to group discrimination using a machine learning approach (see Methods and Supplementary Figure S3). This metric, the negative area of phasic EMG patterns (see Methods and (Poirier et al., 2022; Poirier et al., 2023a), reflects the efficiency of muscle contractions: a higher negativity index indicates more efficient contractions, as the effects of gravity were maximally exploited to conserve energy (Gaveau et al., 2021). In addition, we examined more specific criteria such as amplitude, duration, and frequency (see Supplementary Figure S4). Figure 7A displays the results of the ANCOVA analysis on the index of negativity (Age *×* Task-Type), revealing a significant interaction between age and task factors ( $F_{\left(1,42\right)}$ = 5.48, P = 2.44E-02, ${ƞ}^{2}$ = 0.120) but no Age or Tasks effect (for detailed statistical results, please see Supplementary Table S3). This result indicates that aging differently alters motor strategies for arm movements vs. whole-body movements. Older adults integrate gravity effects to a similar extent as younger ones when performing arm movements (older adults, mean ± SD: −10.7 ± 5.6, 95% CI: [−8.4;−13.0]; younger adults, −11.4 ± 3.6, [−9.8;−13.0]), but to a lesser extent when performing whole-body movements (older adults, −9.7 ± 3.2, [−8.0;−11.5]; younger adults, −15.6 ± 3.3, [−14.1;−17.0]). As recently reported by Poirier et al. (2023b), similar arm results in younger and older adults suggest that the ability to plan optimally efficient movements in the gravity environment remains functional in older adults. The results obtained in whole-body movement tasks (STS/BTS and WBR) could thus suggest that the difference observed between older and younger adults does not reflect a deterioration of the ability to plan movements that are optimally adapted to the gravity environment. Instead, it may suggest a change in movement strategy that compensates for other deteriorated control processes (for example, the loss of muscle mass and force).

We performed a complementary analysis (see Figure 7B) to determine whether every whole-body task showed the same age effect (ANCOVA Age x Whole-Body Tasks). This test did not reveal any interaction effect ( $F_{\left(2,42\right)}$ = 0.77, P = 4.67E-01, ${ƞ}^{2}$ = 0.019), further supporting the interpretation that whole-body tasks are controlled using different motor strategy in older compared to youngers adults (please see Supplementary Table S3 for full analysis).

Previous studies have proposed that the differences observed in whole-body kinematics between older and younger adults could be explained as a strategy maximizing equilibrium maintenance rather than energetic efficiency (Paizis et al., 2008; Casteran et al., 2018). Following this hypothesis, one would predict increasing differences between younger and older adults when the equilibrium constraint increases. In the present experiment, increased equilibrium constraint was produced by increasing the target distance during whole-body reaching movements (WBR D1 vs. WBR D2; alike Casteran et al., 2018). The Age x Whole-body Distances ANCOVA, however, did not reveal such a difference ( $F_{\left(2,42\right)}$ = 2.85, P = 9.91E-02, ${ƞ}^{2}$ = 0.067, please see Supplementary Table S3 for full analysis).

#### 3.1.2 Experimental and modelling results on equilibrium control

**FIGURE 8:**
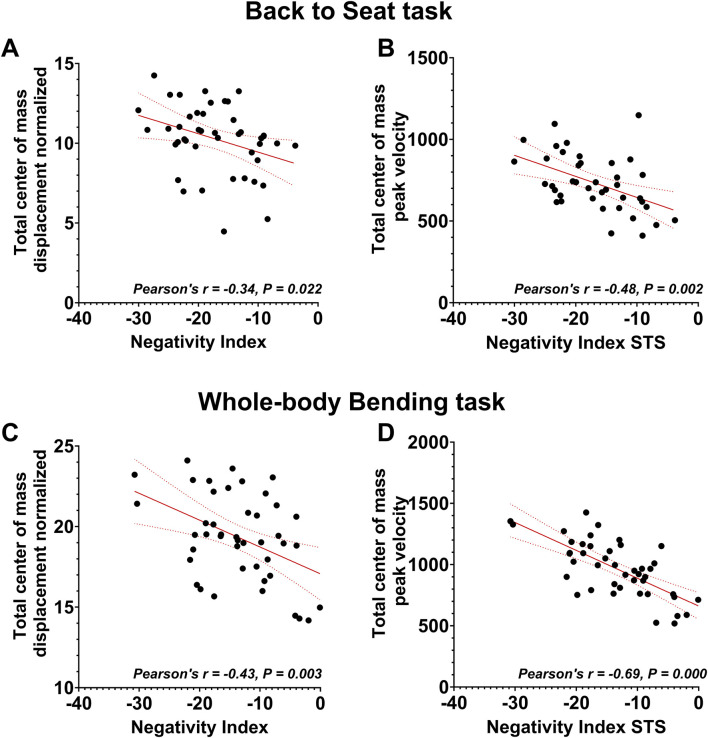
Center-of-mass analyses. Linear relationships between EMG negativity (Vastus Lateralis and Spinal Erector L1) and center-of-mass metrics during whole-body movements. **(A,C)**: Total displacement of the center of mass during back-to-sit **(A)** and bending movements in the whole-body reaching task **(C)**, averaged across distances D1 and D2. **(B,D)**: Peak velocity of the center of mass during back-to-sit **(B)** and bending movements **(D)**, averaged across distances D1 and D2.

Last, we analyzed center of mass kinematics in order to further test the hypothesis that the decreased energetic efficiency observed during whole-body tasks in older adults correspond to a compensation strategy. We first tested whether the negativity of phasic EMGs correlated with kinematic parameters that are related to balance control (the COM displacement, and COM peak velocity, see Figure 8; and see Supplementary Figure S2 for detailed results of the reproduction of the tests conducted by Casteran et al. (2018), Paizis et al. (2008). Phasic EMG negativity during the Back-to-Sit task was found to be significantly correlated with the COM displacement (Pearson’s correlation, P = 2.2E-2, r = -0.343) and the COM peak velocity (Pearson’s correlation, P = 1.9E-3, r = -0.476). It was also significantly correlated for the Whole-Body Bending task with the COM displacement (Pearson’s correlation, P = 3.2E-3, r = -0.435) and with the COM peak velocity (Pearson’s correlation, P = 1.2E-7, r = -0.700). The linear regressions revealed that the more a participant used the effects of gravity, the more and the quicker she/he displaced his/her COM. This suggests that the energy efficient strategy is associated to a more risky strategy in terms of equilibrium control.

**FIGURE 9:**
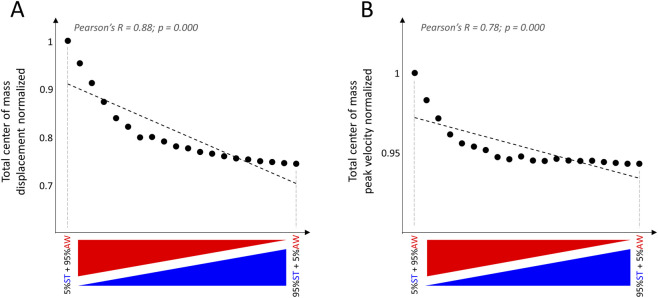
Evolution of center of mass main parameters **(A)** total displacement and **(B)** peak velocity; averaged between distances D1 and D2) across different weightings of the two cost functions.

To formally test this hypothesis, we simulated the total displacement and peak velocity of the center of mass using a composite cost model that combined two criteria: the absolute work of muscle forces (AW) and the sum of joint torques (ST). Prior studies have shown that the AW cost reflects an energetically efficient movement strategy, whereas the ST cost reflects a strategy that prioritizes equilibrium safety (Hilt et al., 2016; Casteran et al., 2018). We conducted a series of simulations in which we systematically varied the relative weighting of these two cost components. Figure 9 illustrates how the resulting kinematic parameters evolved across this weighting spectrum. Consistent with our experimental findings, the simulation results show that increasing the weight of the AW cost—thereby prioritizing energy efficiency over balance safety—leads to larger center-of-mass displacements and higher peak velocities. These findings demonstrate that the kinematic profiles optimal for energy efficiency differ fundamentally from those that prioritize balance maintenance. In other words, energy optimization and equilibrium safety cannot be simultaneously maximized through a single kinematic strategy. This supports the interpretation that older adults may adopt a less energy-efficient strategy during whole-body tasks in order to favor postural stability.

Overall, the results of Experiment 1 confirm that older adults retain the ability to plan energetically efficient arm movements in the presence of gravity (Huang and Ahmed, 2014; Poirier et al., 2020; Healy et al., 2023; Summerside et al., 2024), and strongly suggest that, during whole-body movements, they selects a less energetically efficient but more stable movement strategy. This adaptive selection of motor strategies can be interpreted as a compensation process for other deteriorated ones (e.g., sensory acuity, muscle force).

### 3.2 Experiment 2

In a second experiment, we used a more direct measure of energy expenditure—exhaled gas analysis—to test whether increased equilibrium constraints reduce energy efficiency more significantly in older adults than in younger ones.

**FIGURE 10:**
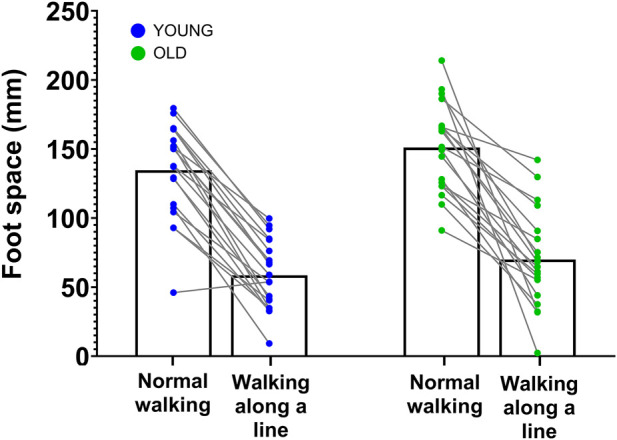
Feet Kinematics. Feet spacing is presented across walking conditions for the two groups. The blue points correspond to the younger participants and the green points correpond to the older participants. Each point corresponds to the mean value of one participant.

We first checked that the tasks were not performed differently depending on the group of participants. As expected, a difference in foot spacing was observed at 2.5 km/h between equilibrium conditions, but not between groups (see Figure 10). A repeated measures ANOVA (Age *×* Equilibrium Conditions) revealed that this walking conditions difference was significant ( $F_{\left(1,37\right)}$ = 174.6, P = 1.39E-15, ${ƞ}^{2}$ = 0.825). The group ( $F_{\left(1,37\right)}$ = 2.83, P = 1.00E-01, ${ƞ}^{2}$ = 0.071) and interaction effects ( $F_{\left(1,37\right)}$ = 0.18, P = 6.76E-01, ${ƞ}^{2}$ = 0.005), on the other hand, were not significant. Similar results were found comparing young participants walking at 4 km/h with older adults walking at 2.5 km/h. We observed a significant difference between walking conditions ( $F_{\left(1,37\right)}$ = 172.67, P = 1.65E-15, ${ƞ}^{2}$ = 0.823) but no group ( $F_{\left(1,37\right)}$ = 2.83, P = 6.65E-02, ${ƞ}^{2}$ = 0.088) or interaction effects ( $F_{\left(1,37\right)}$ = 1.13, P = 2.94E -01, ${ƞ}^{2}$ = 0.029). The results for step length, step variability, and step frequency are presented in Supplementary Figure S5.

#### 3.2.1 Main analysis

**FIGURE 11:**
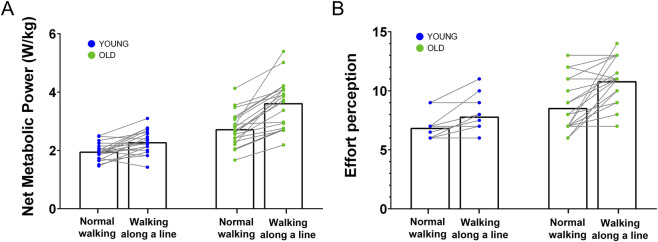
Effort Perception & Net Metabolic Power. Quantification of effort perception **(A)** and Net Metabolic Power **(B)** for both groups. The blue points correspond to the younger participants and the green points correspond to the older participants. Each point in panel B corresponds to the mean value of the Net Metabolic Power during the last minute of each block from which we have subtracted the baseline value calculated between -1min and −30 s.

Figure 11 depicts energy consumption and effort perception results for the two walking blocks. We performed an ANOVA analysis (Age *×* Equilibrium Conditions) to compare groups walking at the same speed (2.5 km/h). We found an Age × Conditions interaction effect for both effort perception ( $F_{\left(1,38\right)}$ = 5.82, P = 2.08E-02, ${ƞ}^{2}$ = 0.133) and net metabolic power ( $F_{\left(1,38\right)}$ = 14.1, P = 5.86E-04, ${ƞ}^{2}$ = 0.270). The equilibrium condition effect was greater in older adults than in younger adults. We also found a significant conditions effect and a significant groups effect. Older adults consumed more energy than younger people for the same walking speed (see Supplementary Table S3 for detailed results). We reproduced this analysis by integrating data from young participants walking at a higher speed (4 km/h). We wanted to match the effort perception of the two groups to test the effect of balance on the same effort perception level. We observed the same trend although the analysis did not reach significance level (Age × Conditions interaction for effort perception: $F_{\left(1,38\right)}$ = 3.07, P = 8.77E-02, ${ƞ}^{2}$ = 0.074 and for net metabolic power: $F_{\left(1,38\right)}$ = 2.74, P = 1.06E-01, ${ƞ}^{2}$ = 0.067, see Supplementary Table S3 for detailed results).

## 4 Conclusion

In this study, we investigated movement efficiency in younger and older adults using two complementary approaches: analysis of muscle activation patterns during arm and whole-body movements (Experiment 1), and analysis of exhaled gas during walking tasks (Experiment 2). The results of Experiment 1 revealed task-dependent, age-related modifications in muscle activation patterns. Specifically, a muscle-based marker of energetic efficiency—previously shown to quantify the extent to which individuals exploit gravity to reduce muscular effort (Gaveau et al., 2021; Poirier et al., 2022; Poirier et al., 2023b). —was significantly reduced in older adults during whole-body movements, but not during arm movements. These findings indicate that the gravity-related efficiency process remains functional in older adults during arm movements, but appears to be downregulated during more complex, full-body tasks.

Consistent with this interpretation, results from Experiment 2 showed that older adults exhibited a greater increase in energetic cost compared to younger adults when walking under conditions that challenged balance. Taken together, these findings suggest that during whole-body movements, older adults may engage a compensatory mechanism that adjusts motor planning to prioritize balance control over energy efficiency.

### 4.1 Maintained efficiency of arm movements in older adults

The metabolic rate is known to influence resource use, body size, rate of senescence, and survival probability (Van Voorhies and Ward, 1999; Brown et al., 2004; DeLong et al., 2010; Strotz et al., 2018). The nervous system has therefore developed the ability to design movement strategies that minimize our every-day efforts (Huang et al., 2012; Selinger et al., 2015; Gaveau et al., 2016; Shadmehr et al., 2016; Morel et al., 2017; Cheval et al., 2018). The present findings confirm the results of previous arm movement studies that proposed a theory according to which motor control takes advantage of gravity effects to save energy (Berret et al., 2008; Crevecoeur et al., 2009; Gaveau and Papaxanthis, 2011; Gaveau et al., 2014; 2016; 2021). Here, we focused on the muscle activation marker of gravity-related energetic-efficiency, i.e., the negativity of phasic EMG. Previous modeling and experimental work demonstrated that this phasic EMG negativity results from an optimal control process that plans efficient arm movements in the gravity field (Gaveau et al., 2021). As reported by Poirier et al. (2023a), we found similar phasic EMG negativity during arm movements in older and younger adults. Thus, arm movements equally optimized gravity effects in younger and older adults. These results align with those of studies that probed progressive motor adaptation to a new environment in older adults. Using locally induced force fields in a robotic environment, these studies revealed that older adults decreased their metabolic costs in a manner similar to younger adults while adapting to new environmental dynamics (Huang and Ahmed, 2014; Healy et al., 2023). Overall, results from arm movement studies advocate for the maintenance of the ability to optimally integrate environmental dynamics and plan arm movements that are energetically efficient in older adults.

### 4.2 Whole-body movements also harvest gravity effects to save energy

Current results also extend the current knowledge on the planning of energetically efficient movements to more global movements, both in younger and older adults. They reveal that deactivating muscles below the tonic level required to compensate for external dynamics is important not only for controlling focal arm movements but also for whole-body movements. Using a combination of modeling and experimental work, previous studies demonstrated that healthy participants move their arms following trajectories and using muscular patterns that save energy in the gravity environment (Berret et al., 2008; Crevecoeur et al., 2009; Gaveau and Papaxanthis, 2011; Gaveau et al., 2014; 2016; 2021). To isolate gravity effects, most studies focused on one-degree-of-freedom arm movements. Although those studies allowed us to clearly demonstrate how motor planning integrates gravity effects into motor planning, one-degree-of-freedom movements are hardly representative of the rich and complex human movement repertoire. The present study extends the optimal integration of gravity effects theory to movements that are more ecological—that is, multi-joint and whole-body actions resembling those performed in everyday life.

### 4.3 Decreased efficiency of whole-body movements in older adults

Contrary to focal arm movements, we observed a strong age effect during global movements that engaged the entire body (i.e., sit-to-stand/back-to-sit, whole-body reaching movements and walking). Specifically, the negativity of phasic EMG was significantly reduced in older compared to younger adults. This suggests that whole-body movements are less energetically efficient in older adults than in younger ones, adding to the general result that global movements are more energy-demanding for older adults compared to younger adults (Didier et al., 1993; Hortobagyi et al., 2003; 2011; John et al., 2009). Previous kinematic studies suggested that older adults favor movement strategies that maximize balance maintenance rather than energy efficiency (Paizis et al., 2008; Casteran et al., 2018). However, age differences observed during whole-body movements may also be interpreted as an inability to save energy when coordinating complex movements (Goodpaster et al., 2006; Vernazza-Martin et al., 2008; Quinlan et al., 2018; Henry and Baudry, 2019). Here, contrasting results from arm and whole-body movements in the same participants, we provide support for a compensation process that adapts movement strategy in older adults, rather than a deterioration of the ability to optimally coordinate whole-body movements. Since arm movements revealed that older participants maintained the ability to plan energetically efficient movements, altered whole-body movement may be explained as an adaptation of movement strategy rather than deteriorated motor planning. Moreover, we found that decreased efficiency was associated with decreased center-of-mass displacement and speed, i.e., less instability. This further suggests that decreased efficiency in older adults may represent a compensation process that trades efficiency with equilibrium maintenance. This could be explained as an optimal motor planning process that minimizes a composite cost function; i.e., energy and instability. It has been proposed that the central nervous system combines different costs–related to energy, precision, or duration, for example,–when planning a movement (Liu and Todorov, 2007; Gielen, 2009; Mombaur et al., 2010; Berret et al., 2011; Vu et al., 2016; Healy et al., 2023; Poirier et al., 2023b; Tanis et al., 2023). In older adults, this combination would increase the relative weighting of the instability (equilibrium) cost and decrease the relative weighting of the energetic cost. Future work may use this framework to probe age-related motor adaptation.

### 4.4 Age-related compensation in the brain

In the sensorimotor field, following the consensus that aging is associated with increased activation and increased spatial recruitment, numerous studies have attempted to establish a correlation between brain activation and behavioral performance in older adults (Ward, 2006; Seidler et al., 2010; Poirier et al., 2021; Fettrow et al., 2021). This literature has not reached a consensus on the neural changes underlying compensatory mechanisms in the aging brain. Several studies reported a positive correlation (Cassady et al., 2020a; Clark et al., 2014; Harada et al., 2009; Heuninckx et al., 2008; Holtzer et al., 2015; Jor’dan et al., 2017; Larivière et al., 2019; Mattay et al., 2002; Spedden et al., 2019), and as many reported no correlation or even a negative correlation (Riecker et al., 2006; Ward et al., 2008; Loibl et al., 2011; Bernard and Seidler, 2012; Holtzer et al., 2016; Hawkins et al., 2018; Cassady et al., 2019; Fernandez et al., 2019; Cassady et al., 2020b). Building on the theoretical framework proposed by Krakauer et al. (2017), we previously argued that a major reason for the ongoing lack of consensus regarding age-related compensatory mechanisms is methodological: many prior studies have focused on brain activation while relying on rudimentary behavioral paradigms, potentially conflating deterioration with compensation (Poirier et al., 2021). In contrast, behavioral paradigms specifically designed to isolate distinct motor control processes—such as those employed in the present study—may offer a more precise means of disentangling compensatory strategies from age-related decline. From this perspective, the present study addresses a key limitation of prior work by using behavioral paradigms that more precisely isolate motor control processes, thereby providing a necessary behavioral framework for interpreting age-related compensatory mechanisms, including those investigated at the neural level. We suggest that future work examining compensatory processes at the neural level may benefit from adopting such behavioral approaches.

### 4.5 Age-related changes in effort and energy consumption

During the walking task, we observed a more pronounced increase in both perceived effort and energy expenditure (via exhaled gas analysis) in older adults compared to younger adults as balance challenges intensified. Previous studies have suggested that older adults prioritize movement strategies that enhance balance safety (Jensen et al., 2001; Li et al., 2001; Mille et al., 2003; Paizis et al., 2008; Casteran et al., 2018; Rathore et al., 2022). However, the differences seen in whole-body movements between age groups may also indicate an age-related difficulty in conserving energy during complex movements (Goodpaster et al., 2006; Vernazza-Martin et al., 2008; Quinlan et al., 2018; Henry and Baudry, 2019). Our findings suggest that increased energy expenditure is associated with higher balance demands, implying a compensatory mechanism that adjusts movement strategies in response to these constraints. Our study confirms the strategy shift that occurs with age, reducing the emphasis on effort minimization in favor of balance maximization. Interestingly, recent research has reported that the increased energy cost of walking in older participants is primarily linked to neuromuscular mechanisms and changes in connective tissue (Boyer et al., 2023). Here, we demonstrate that there is also a “strategic” component, potentially aimed at minimizing the risk of falling, even if it means consuming more energy.

### 4.6 Walking speed as a factor

Walking speed was an important parameter in Experiment2. We selected a relatively low speed (2.5 km/h) to ensure that all older participants could complete the task. However, previous literature has reported that older individuals have a greater perception of effort and higher energy costs than younger individuals performing the same tasks (Didier et al., 1993; Hortobagyi et al., 2003; 2011; John et al., 2009). Therefore, we asked younger participants to also perform the walking task at a higher speed (4 km/h, after preliminary tests, to match the effort perception). In addition to the interaction effect showing that older adults perceive more effort than younger people when balance constraints increase at the same speed, the results also show an age-related effect. This confirms that older adults perceive more effort for the same task. However, comparing at the same speed is still relevant, as older participants face the same everyday challenges as the young ones. For example, the time allowed to cross a crosswalk does not depend on the age of the person crossing (Asher et al., 2012). Future studies might consider testing participants at their spontaneous walking speed, as this differs with age (Himann et al., 1988; Malatesta et al., 2003).

### 4.7 Role of physical and cognitive fitness in age-related compensation

Physical and cognitive fitness may influence the extent to which older adults favor stability over energetic efficiency. It is well-known that physical and cognitive fitness significantly impact functional mobility in older adults (Zhao et al., 2014; Marusic et al., 2018; Wickramarachchi et al., 2023). One could speculate that physical and cognitive fitness are inversely related to the level of physical and cognitive deterioration. For example, muscle force and sensory integration are crucial for controlling balance. The more deteriorated they are, the greater the need for compensatory processes to adapt movement control to the participant’s capacities. Future research should account for variations in physical and cognitive fitness to better understand their role in the development of compensatory mechanisms.

### 4.8 Simple mono-articular vs. complex multi-articular arm movements

Another aspect that needs to be highlighted here is the choice of our arm task. Using this very same task, the results from two previous studies also support the preservation of arm movement efficiency in older adults (Poirier et al., 2020; Poirier et al., 2023a). It is important to emphasize, however, that the preserved efficiency observed here is specific to a simple, single-joint pointing task requiring minimal coordination. Numerous studies have documented age-related differences in proprioception, coordination, and movement variability across a broader range of upper-limb actions, and our findings should therefore not be interpreted as evidence of a general preservation of arm motor planning in aging. Rather, they indicate that, under constrained mechanical conditions, older adults remain capable of producing movement solutions that are energetically efficient. Other studies that used multi-degree-of-freedom arm movements to examine motor adaptation to an externally imposed force field have also found that, like younger adults, older adults retain the ability to produce energetically efficient movements (Healy et al., 2023; Summerside et al., 2024). Consistent with this view, recent work has shown that while feedback-based stabilization and final movement accuracy decline with age, feedforward control mechanisms supporting movement initiation and coordination can remain preserved even in older adults (Matthijs et al., 2025). The present mono-articular results may thus generalize to other types of arm movements, although such extrapolation remains speculative for now. In young adults, the efficient integration of gravity effects to save energy has been demonstrated with varied arm movements, such as single or muti-degree of freedom pointing movements, drawing movements, reach to reach-to-grasp movements, or arm movements that transport a hand-grasped object (Papaxanthis et al., 1998; 2005; Le Seac’h & McIntyre, 2007; Berret et al., 2008; Crevecoeur et al., 2009; Gaveau et al., 2011; Gaveau and Papaxanthis, 2011; Yamamoto and Kushiro, 2014). Future work may test whether the present conclusions in older adults extend to more complex and functional arm movements.

### 4.9 Limitations

As mentionned in some parts of the discussion section, several limitations should be considered when interpreting the present findings. Here we synthetize these limitations. First, the behavioral tasks were designed to isolate specific motor control processes, but they necessarily simplify real-world walking and may not capture all aspects of everyday locomotion. Second, measures of walking efficiency and muscle activity rely on modeling and measurement assumptions that may influence quantitative estimates. Third, while the study focuses on healthy older adults, the extent to which these findings generalize to more heterogeneous aging populations or to clinical groups remains to be determined. Finally, although we interpret the observed patterns as consistent with a trade-off between stability and efficiency, alternative explanations—such as increased co-contraction, reduced selective muscle activation, or sensory decline—cannot be fully ruled out. Future work will try to specifically adress these limitations.

In conclusion, by probing a specific motor control process and quantifying the effort involved, the present study provides a set of behavioral results that support the interpretation of a compensatory process that counterbalances other deteriorated processes in older adults. Probing age effects on specific sensorimotor control processes may help disentangle compensation from deterioration processes that occur through healthy aging (Poirier et al., 2021). We believe that understanding compensation at a behavioral level is an important step toward pinpointing its neural underpinning (Krakauer et al., 2017) and, later, preventing unhealthy aging (Baltes and Baltes, 1990; Martin et al., 2015; Zhang and Radhakrishnan, 2018).

## Data Availability

The datasets presented in this study can be found in online repositories. The names of the repository/repositories and accession number(s) can be found below: Data are available online: https://doi.org/10.5281/zenodo.15874180. Scripts and code are available online: https://doi.org/10.5281/zenodo.15922728.
