# Supplementary material for: Comparing arm to whole-body motor control disambiguates age-related deterioration from compensation
Source: Front Aging. 2026 Mar 18;7:1715723. doi: 10.3389/fragi.2026.1715723 (PMC13039035; doi:10.3389/fragi.2026.1715723)
Supplement: Supplementary file 1 [file DataSheet1.pdf]

## Supplementary information

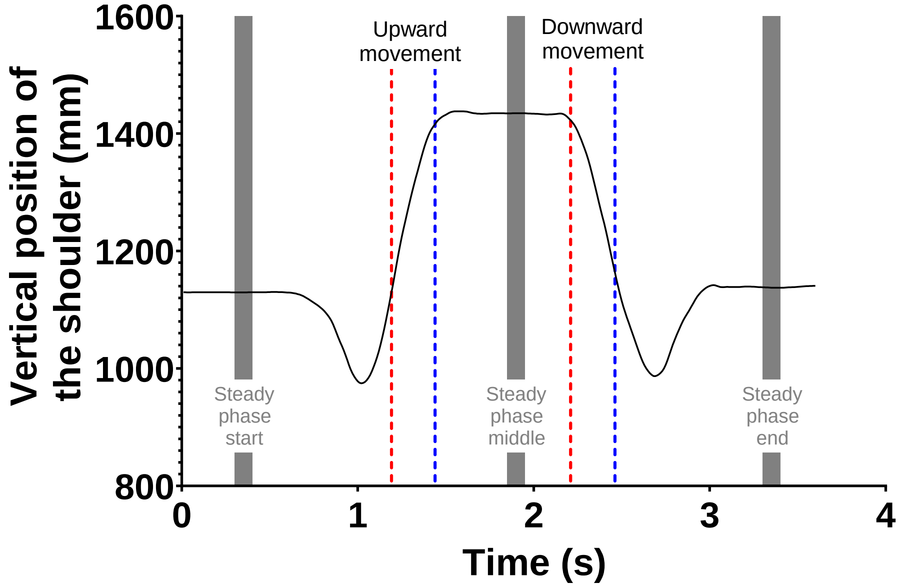


**Supplementary Figure 1. Detection of movement start and end.** This panel shows the recording of two successively opposite fast sit-to-stand / back-to-sit movements. The black trace represents the position of a shoulder marker through time. Rest position is collected during steady phases, before and after each movement (ascending or descending). Based on the data obtained in steady phases, a recursive algorithm automatically defined movement onset and offset as the moments when the displacement rose above or fell below a threshold corresponding to 5% and 95% of the total movement amplitude, respectively.

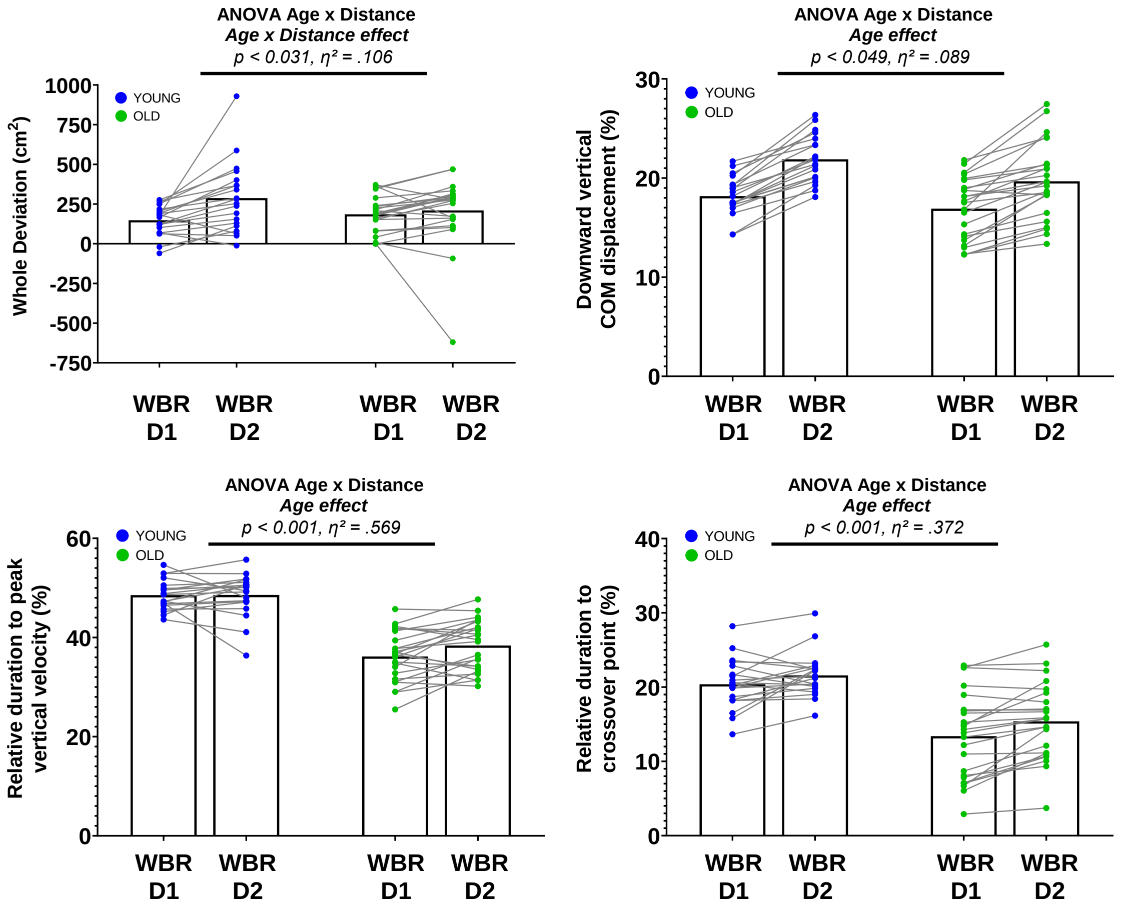


**Supplementary Figure 2.** Reproduction of the analyses of Paizis et al. (2008) (panels **A** and **B**) and Casteran et al. (2018) (panels **C** and **D**). Both studies showed age-related differences in motor skills during balance tasks (downward whole-body reaching tasks).

**Paizis et al. (2008)**. We computed the two main criteria used by Paizis et al. (2008): the whole deviation and the downward vertical center of mass (CoM) displacement. The whole deviation was computed using a trapezoidal numerical integration from Matlab [Mat, 2021] to obtain the area between the actual trajectory from the wrist and the straight path between movement start and end. The downward vertical displacement was computed on the CoM using the mathematical seven-segment model of the body detailed in the manuscript between movement start and end and was normalized by the height of the participant. Paizis et al. (2008) only tested one distance. We observe an Age × Distance interaction, whereas Paizis et al. (2008), with only one distance, only observe an Age effect. We observe the same Age effect as they did for the vertical CoM displacement, with the younger participants having more displacement than the older ones. The results we found are in direct agreement with those obtained by the authors.

**Casteran et al. (2018)**. We computed the two main criteria used by Casteran et al. (2018): the relative duration to peak vertical velocity of the CoM and the relative duration to crossover point. The relative duration to peak vertical velocity of the CoM was obtained using the mathematical seven-segment model of the body detailed in the manuscript between movement start and end. The crossover point between

the anteroposterior and vertical velocities was obtained by decomposing the CoM velocity profile into its Antero-Posterior (ΔAP trajectory) and Vertical (ΔV) components. The point of interest is the moment when the vertical component surpasses the antero-posterior one. Casteran et al. (2018) tested the same two distances. We observe an Age effect for the relative time to peak vertical velocity, as Casteran et al. (2018) did, suggesting a vertical peak velocity occurring earlier for older participants. We also found an Age effect for the crossover point between vertical and antero-posterior components of the CoM velocity, while Casteran et al. (2018) suggested that – but did not directly test – there may exist an interaction effect Age x Distances. The present results do not validate the supposed interaction effect. Distance does not seem to more strongly affect the movements of older adults compared to younger adults.

| Subjects | | ARM | STS/BTS | | | | WBR D1 | | | | WBR D2 | | | |
|---|---|---|---|---|---|---|---|---|---|---|---|---|---|---|
| | | DA | VL | | ESL1 | | VL | | ESL1 | | VL | | ESL1 | |
| | | Right | Right | Left | Right | Left | Right | Left | Right | Left | Right | Left | Right | Left |
| Young | 1 | X | X | X | X | NO | X | X | X | NO | X | X | X | NO |
| | 2 | X | X | X | X | X | X | X | Slow | Slow | X | X | X | X |
| | 3 | X | X | X | X | NO | X | X | X | NO | X | X | X | NO |
| | 4 | X | X | X | X | NO | X | X | X | NO | X | X | X | NO |
| | 5 | X | X | X | X | NO | X | X | X | NO | X | X | X | NO |
| | 6 | X | X | NO | NO | NO | X | X | X | NO | X | X | X | NO |
| | 7 | X | X | X | X | NO | X | X | X | NO | X | Slow | Slow | NO |
| | 8 | X | X | X | X | X | X | X | X | X | X | X | X | X |
| | 9 | X | X | X | X | X | X | Slow | X | Slow | X | X | X | X |
| | 10 | X | X | X | X | X | X | X | X | X | X | X | X | X |
| | 11 | X | X | X | X | X | X | X | X | X | X | X | X | X |
| | 12 | X | X | X | X | X | X | X | Slow | Slow | X | X | X | X |
| | 13 | X | X | NO | X | X | Fast | NO | X | X | X | X | X | X |
| | 14 | X | X | X | X | X | X | X | X | X | X | X | X | X |
| | 15 | X | X | X | X | X | X | X | X | X | X | X | X | X |
| | 16 | X | X | X | X | Slow | X | X | X | X | X | X | X | X |
| | 17 | X | X | X | X | X | X | X | NO | NO | X | X | X | X |
| | 18 | X | X | X | X | X | X | X | Slow | Slow | X | X | Slow | Slow |
| | 19 | X | X | X | X | X | X | X | X | X | X | X | X | X |
| | 20 | X | X | X | X | X | X | X | X | X | X | X | X | X |
| Old | 1 | X | X | X | X | Fast | X | X | X | X | X | Fast | X | X |
| | 2 | X | X | X | X | X | X | X | X | X | X | X | X | X |
| | 3 | X | X | X | X | X | X | X | X | X | X | X | X | X |
| | 4 | X | X | X | X | X | X | X | X | NO | X | Slow | Slow | NO |
| | 5 | X | X | X | X | X | NO | Slow | Slow | Slow | NO | X | X | X |
| | 6 | X | X | X | X | X | Slow | X | NO | NO | X | X | NO | NO |
| | 7 | X | X | X | X | X | X | X | Slow | X | X | X | X | X |
| | 8 | X | X | X | X | X | X | X | X | X | X | X | X | X |
| | 9 | X | X | NO | X | X | Fast | NO | X | X | Fast | NO | X | X |
| | 10 | X | X | X | X | X | X | NO | X | X | X | NO | X | X |
| | 11 | X | NO | NO | X | X | NO | NO | X | X | NO | NO | X | X |
| | 12 | X | Slow | X | NO | X | X | X | NO | X | X | X | NO | X |
| | 13 | X | X | X | X | X | X | NO | X | X | Fast | NO | X | X |
| | 14 | X | X | X | X | X | X | X | X | X | X | X | X | X |
| | 15 | X | X | X | X | X | X | X | X | X | X | X | X | X |
| | 16 | X | X | X | X | X | X | X | X | X | X | X | X | X |
| | 17 | X | X | X | X | X | X | X | X | X | X | X | X | X |
| | 18 | X | X | X | Slow | X | X | NO | X | X | X | X | NO | NO |
| | 19 | X | X | X | X | X | X | X | X | X | Fast | Fast | Fast | Fast |
| | 20 | X | X | X | X | X | X | X | X | X | X | X | X | X |
| | 21 | X | X | X | X | X | X | X | X | X | X | X | X | X |
| | 22 | X | X | X | X | X | X | X | X | X | X | X | X | X |
| | 23 | X | X | X | X | X | Slow | Slow | Slow | Slow | X | X | X | X |
| | 24 | X | X | X | X | X | X | X | X | X | X | X | X | X |

**Supplementary Table 1.** Some of the EMG recordings exhibited aberrant values. This table reports the quality of EMG recordings for the main antigravity muscles in each participant (numbered on the left), and each task, i.e. DA (Deltoid Anterior head) during focal arm movements, and VL (Vastus Lateralis) and ESL1 muscles (Erector Spinae at Lumbar 1 level) during global whole-body movements. “X”: all trials were correct. “Fast”: some of the fast trials, maximum 4/12, have been excluded. “Slow”: some of the slow trials, maximum 2/6, have been excluded. “NO”: too many trials exhibited aberrant values and, therefore, the muscle was not used for the analyses presented in this manuscript. STS/BTS: Sit-to-stand/Back-to-sit. WBR D1: Whole body reaching near target. WBR D2: whole body reaching far target.

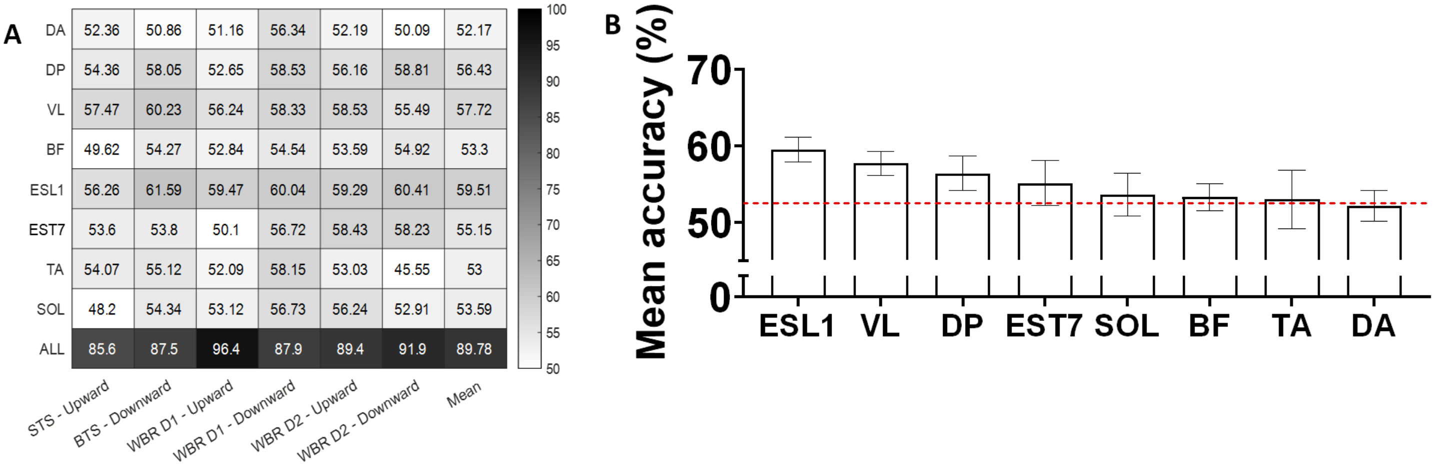


**Supplementary Figure 3.** A. Heatmap representing the accuracy of the LDA algorithm to discriminate young from older adults using phasic EMGs recorded during the tasks mobilizing the entire body. The eight first lines correspond to the accuracy of each individual muscle. The last line corresponds to the accuracy of taking all muscles simultaneously (DA: Anterior deltoid, DP: Posterior deltoid, VL: Vastus Lateralis, BF: Biceps Femoris, ESL1: Erector Spinae in L1, EST7: Erector Spinae in T7, TA: Tibialis Anterior, SOL: Soleus). The first six columns correspond to the six whole-body tasks of the experiment (STS = Sit To Stand; BTS = Back To Sit; WBR D1 = Whole Body Reaching near target; WBR D2 = Whole body reaching far target), the last column corresponds to the average accuracy of the six tasks for each muscle. This analysis has been conducted to highlight which muscles are important for discrimination. We can see that those antigravity muscles (muscles that act against gravity, in our case, the VL and the ESL1) contain relevant information as they reach the highest scores. B. Bargraph representing the accuracy ± SD of the LDA algorithm for each individual muscle in ascending order (DA: Anterior deltoid; DP: Posterior deltoid; VL: Vastus Lateralis; BF: Biceps Femoris; ESL1: Erector Spinae in L1; EST7: Erector Spinae in T7; TA: Tibialis Anterior; SOL: Soleus). We can see that those antigravity muscles (muscles that act against gravity, in our case, the VL and the ESL1) contain the most relevant information as they reach some of the highest values. Error bars correspond to the standard error across a five-fold cross-validation.

**Motivation**. Here we were interested in comparing arm movement and whole-body movement control because the scientific literature has reported that the control of whole-body movements changes with age, while the control of arm movements does not (Casteran et al., 2018; Paizis et al., 2008; Poirier et al., 2020, 2024; Vernazza-Martin et al., 2008). Focusing on a limited number of muscles is problematic, as we risk probing muscles whose activation patterns may not vary with age. To ensure that our restrictive theory-driven analysis provides meaningful results, we verified that our cherry-picked muscles truly conveyed information about age-related modifications of whole-body movement control. To this aim, we employed machine learning analyses that quantified how much each muscle activation was altered by age. This allowed us to verify that we were indeed focusing on muscles that discriminated movement control between younger and older adults.

Our rationale was the following: if the algorithm can successfully separate the data of younger and older adults, using antigravity muscle activation patterns, this would demonstrate that important information is contained in those muscles regarding age-related modifications of movement control. For more details on similar use and operation of machine learning algorithms on EMG signals, please see (Chambellant et al., 2024; Thomas et al., 2023; Tolambiya et al., 2011). Here we present the results of a Linear Discriminant Analysis (LDA, Johnson & Wichern, 1988) but we verified that we obtained similar conclusions with two other algorithms, namely the Quadratic Discriminant Analysis (QDA, Cover, 1965) and the Support Vector Machine (SVM, Vapnik and Lerner, 1965).

The Machine Learning analysis indeed revealed that antigravity muscles contained important information, allowing us to separate age groups with the highest classification accuracies (see Figure 8 for results regarding

LDA accuracy). The vastus lateralis (VL) and the spinal erectors on L1 (ESL1) achieved the best classification accuracies of 57.72% and 59.51% respectively (considering that these classifications are significantly better than chance if they are above 52.5% according to a fairness test). The main results presented here are therefore quantitatively based. They originate from analyses of the muscles that show the most information to distinguish younger from older adults during whole-body movement. Other muscles, such as DP or EST7, also exhibit reasonably good classification accuracies. This is not unexpected as humans and animals are known to control their varied muscles in a synergistic manner (Berret et al., 2009; d'Avella et al., 2006; Tresch et al., 1999), and even the slightest alteration of movement strategy may require modifying the activation of several muscles.

**Supplementary Method 1**

**Musculoskeletal System Modeling**

As in (Hilt et al, 2016; Casteran et al, 2018) we modeled the dynamics of the musculoskeletal system using a series of articulated rigid bodies, with the joints primarily moving in the sagittal plane (six key joints, excluding the head segment). Using the classical Lagrangian formalism, the dynamics of the entire system can be expressed as:

$$\tau = M(\theta)\ddot{\theta} + C(\theta, \dot{\theta})\dot{\theta} + G(\theta)$$

In this equation:

- $\theta = (\theta_1, \theta_2, \theta_3, \theta_4, \theta_5, \theta_6)^T$ represents the vector of joint angles.
- $\tau = (\tau_1, \tau_2, \tau_3, \tau_4, \tau_5, \tau_6)^T$ denotes the vector of resultant muscle torques.
- $M(\theta)$ is the inertia matrix.
- $C(\theta, \dot{\theta})$ contains the Coriolis and centripetal terms.
- $G(\theta)$ accounts for the gravitational torques.

As previously done, we neglected viscous friction and the elastic properties of tissues in this model and we employed a recursive Newton-Euler algorithm to compute movement dynamics, to enhance computational efficiency. We utilized the planar spatial vector formalism developed by R. Featherstone. The implementation of these algorithms in MATLAB, provided by the author and freely available online, was used in our simulations.

To account for the low-pass filter properties of skeletal muscles, we incorporated a simple model of muscle dynamics. In line with previous studies, we assumed that the control measure was the time derivative of muscle torque, modeled as a first-order low-pass filter. This approach was chosen to capture the smoothness observed in torque and acceleration profiles during human movement, particularly at the onset and conclusion of transient motions. Combined with the limb dynamics, this formed the control system, hereafter referred to as (Σ).

**Optimal Control Methodology**

The goal of optimal control in this context is to determine the movement trajectory that minimizes a specific optimality criterion J, which is defined based on task constraints and the equations of motion, including subject-specific anthropometric data. Mathematically, the problem can be described as follows: find an admissible control u and the corresponding admissible state trajectory q of the system Σ, which connects an initial point A to a final point B within a specified time T, while minimizing the cost function J. A control or trajectory is deemed admissible if it satisfies the control and state constraints throughout the entire movement duration. The state trajectory q encompasses the position, velocity, and acceleration of the six joint angles over time, represented as:

$$q = (\theta_i, \dot{\theta}_i, \ddot{\theta}_i)_{1 \le i \le 6}$$

The system's dynamics involve a high-dimensional (18-dimensional) state space, with non-linear dynamics and potentially non-quadratic cost functions, making the problem challenging to solve. To address these challenges, we employed numerical methods (detailed below) that provided accurate convergence properties. The combined limb and muscle dynamics formed a fully actuated control system Σ, which could be linearized through feedback. By controlling the derivative of the angular acceleration vector instead of the derivative of muscle torque, we reduced the non-linear dynamics to a linear form. Specifically, we used the angular jerk, defined as the rate of change of angular acceleration u, as an abstract control variable:

$$u = (\dddot{\theta}_i)_{1 \le i \le 6}$$

We selected two cost functions based on previous investigations:

(1) a combination of absolute work of torques and angular jerk ("reach-efficient"; Hilt et al, 2016),
$C_{AJ} = \int_0^T \sum_{i=1}^{6} \dddot{\theta}_i^2 \, dt$ ; $C_{AW} = \int_0^T \sum_{i=1}^{6} \left|\dot{\theta}_i . \tau_i\right| dt$

(2) the sum of torques ("balance-efficient"; Hilt et al, 2016)

$$C_{ST} = \int_0^T \sum_{i=1}^{6} \tau_i^2 \, dt$$

During our simulations, the initial point A, and time T were matched for an average subject (height: 170 cm, weight: 70 kg) based on experimentally recorded mean initial postures and movement durations. The target position, which specifies the final position to be reached within a 1 cm accuracy, was determined from experimental values and included two distances: 15% [D1] and 30% [D2] of arm length. Since the subjects began and ended their movements in a quasi-static equilibrium state, we assumed zero angular velocity and acceleration at both the start and end of the movements. Anthropometric parameters, such as moments of inertia and segment masses, were derived from documented sources (Winter, 1990), and horizontal surface-shoulder distances were based on experimental mean values, accounting for inter-individual physical differences and experimental variations. To account for biological joint limits, we constrained each joint angle within a realistic range, based on the maximum and minimum joint angles observed across all subjects and trials. Velocities, accelerations, and jerks were also constrained to relatively large values, and we verified post hoc that these boundary values were never reached during simulations.

To ensure whole-body equilibrium, we added a path constraint that required the center of pressure (CoP) and the vertical projection of the center of mass (CoM) to remain within the base of support throughout the movement. The CoP location was calculated using muscle torques and forces derived from inverse dynamics, considering the control of angular jerk during simulations. The anterior-posterior (A-P) CoP position was computed using the formula described by (Martin et al, 2006), based on the fundamental principle of static equilibrium applied to the feet (which were assumed to be fixed, as required by the real experiment). Whole-body CoM position was determined using Winter's tables (Winter, 1990), which also provided the CoM position of each moving segment in the sagittal plane. The A-P CoP location had to remain within the bounds defined by the maximum CoP displacement observed experimentally across subjects and conditions, specifically between −0.05 m and 1.5 times the foot length (from the malleolus marker to the fifth metatarsal). This constraint was imposed as a nonlinear path constraint in the optimal control problem formulation.

To solve the optimal control problem, we employed a direct transcription technique, transforming it into a nonlinear programming (NLP) problem with constraints. We used the Gaussian pseudo-spectral method to convert the continuous optimal control problem into a discrete one and utilized the MATLAB software implementation GPOPS (Rao et al, 2010). The resulting NLP problem was then solved using the established numerical software SNOPT (Gill et al, 2002).

**Analysis of the Model's Results**

To compare the model's results with actual data, we calculated the same parameters that had previously been derived from the tested subjects' center of mass: normalized total center of mass displacement and peak center of mass velocity. These parameters were selected because the center of mass is a key factor in distinguishing between the two optimal models, as noted in previous studies (Hilt et al., 2016; Casteran et al., 2018). Our analysis of the real data also revealed a strong correlation between these parameters and the negativity index (Figure 8).

Furthermore, to investigate the relationship between the two optimal cost functions and the negativity index, we ran a series of simulations. In these simulations, the weightings of the two cost functions were adjusted gradually in inverse proportion, as detailed in Supplementary Table XX. In Figure XX, the first value on the left corresponds to the result of Simulation 1, where the AW cost is minimally weighted (5%) and the ST cost is maximally weighted (95%). Conversely, the last value on the right represents the result of Simulation 20, where the AW cost is maximally weighted (95%) and the ST cost is minimally weighted (5%).

| Task | | Direction | Group | Mean (s) ± SD (s) |
|---|---|---|---|---|
| ARM | Tonic | Upward | OLD | 6,104± 2,8 |
| | | | YOUNG | 3,907± 1,4 |
| | | Downward | OLD | 6,395± 3,6 |
| | | | YOUNG | 3,544± 1,6 |
| | Fast | Upward | OLD | 0,221± 0,08 |
| | | | YOUNG | 0,210± 0,08 |
| | | Downward | OLD | 0,249± 0,10 |
| | | | YOUNG | 0,213± 0,08 |
| STS/BTS | Tonic | Upward | OLD | 2,144± 0,7 |
| | | | YOUNG | 1,698± 0,5 |
| | | Downward | OLD | 2,858± 1,0 |
| | | | YOUNG | 2,141± 0,6 |
| | Fast | Upward | OLD | 0,357± 0,09 |
| | | | YOUNG | 0,267± 0,05 |
| | | Downward | OLD | 0,396± 0,11 |
| | | | YOUNG | 0,279± 0,04 |
| WBR D1 | Tonic | Upward | OLD | 4,009± 1,2 |
| | | | YOUNG | 3,106± 1,0 |
| | | Downward | OLD | 3,375± 0,9 |
| | | | YOUNG | 2,739± 0,7 |
| | Fast | Upward | OLD | 0,521± 0,11 |
| | | | YOUNG | 0,419± 0,05 |
| | | Downward | OLD | 0,458± 0,07 |
| | | | YOUNG | 0,389± 0,05 |
| WBR D2 | Tonic | Upward | OLD | 4,083± 1,3 |
| | | | YOUNG | 3,276± 0,9 |
| | | Downward | OLD | 3,594± 1,2 |
| | | | YOUNG | 2,747± 0,8 |
| | Fast | Upward | OLD | 0,529± 0,10 |
| | | | YOUNG | 0,445± 0,06 |
| | | Downward | OLD | 0,525± 0,09 |
| | | | YOUNG | 0,442± 0,05 |

**Supplementary Table 2.** Mean movement durations ± (SD) are presented for each task, movement direction, movement speed and age-group. STS/BTS = Sit-to-Stand / Back-to-Sit; WBR D1 = Whole-Body Reaching near target; WBR D2 = Whole-Body reaching far target.

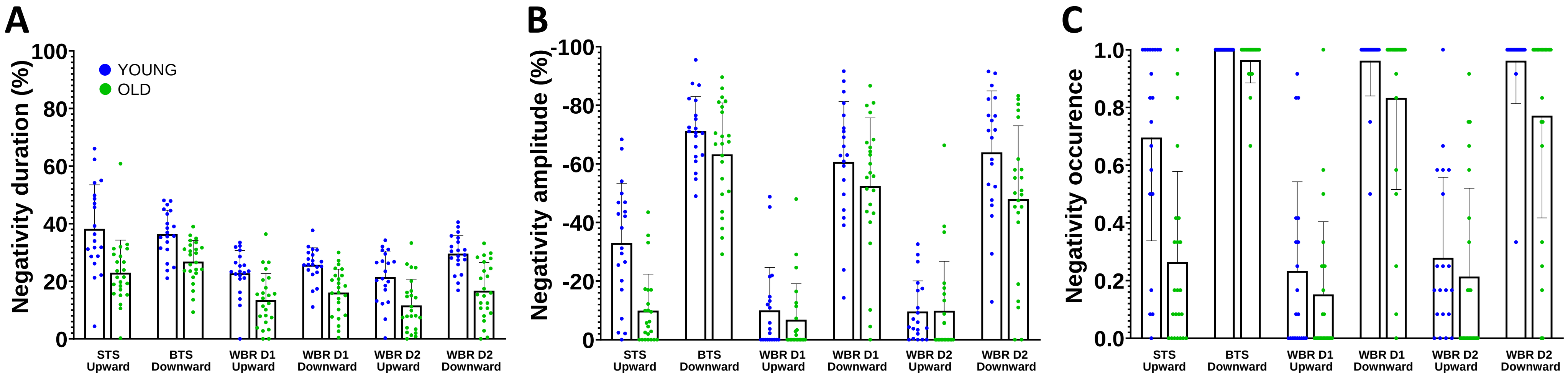


**Supplementary Figure 4**. Mean ± SD negativity quantification for all tasks and groups (STS: Sit-to-stand, BTS: Back-to-sit, WBR: Whole-body-reaching, D1: Short distance=15% of the height of the subject, and D2: Long distance=30% of the height of the subject). Quantification was carried out using three criteria: negativity duration (panel A), negativity amplitude (panel B), and negativity occurrence (panel C) on the antigravity muscles (vastus lateralis and the erector spinae). The blue points correspond to the younger participants, and the green points correspond to the older participants. Each point corresponds to the mean value across trials and antigravity muscles.

**Experiment 1**

| | Effects | F | p-value | Effect size (partial $\eta^2$) |
|---|---|---|---|---|
| **A. Movement duration** / **ANOVA Age x Type of Tasks** | Type of Tasks effect | 433 | 9.38E-24 | 0.911 |
| | Age effect | 14.5 | 4.58E-04 | 0.256 |
| | Age x Type of Tasks effect | 20.0 | 5.72E-05 | 0.322 |
| **B. Negativity Index** / **ANCOVA Age x Whole-body tasks** | Type of Tasks effect | 0.53 | 4.70E-01 | 0.013 |
| | Age effect | 0.54 | 4.68E-01 | 0.013 |
| | Age x Type of Tasks effect | 5.48 | 2.44E-02 | 0.120 |
| **C. Negativity Index** / **ANCOVA Age x Type of Tasks** | Whole-body Tasks effect | 0.78 | 9.30E-01 | 0.002 |
| | Age effect | 4.50 | 4.00E-02 | 0.103 |
| | Age x Whole-body Tasks effect | 0.77 | 4.67E-01 | 0.019 |
| **D. Negativity Duration** / **ANCOVA Age x Whole-body tasks** | Whole-body Tasks effect | 0.32 | 7.20E-01 | 0.009 |
| | Age effect | 21.5 | 4.54E-05 | 0.374 |
| | Age x Whole-body Tasks effect | 2.49 | 8.99E-02 | 0.065 |
| **E. Negativity Amplitude** / **ANCOVA Age x Whole-body tasks** | Whole-body Tasks effect | 0.63 | 5.37E-01 | 0.017 |
| | Age effect | 1.16 | 2.80E-01 | 0.031 |
| | Age x Whole-body Tasks effect | 0.72 | 4.89E-01 | 0.020 |
| **F. Negativity Frequency** / **ANCOVA Age x Whole-body tasks** | Whole-body Tasks effect | 0.39 | 6.75E-01 | 0.011 |
| | Age effect | 3.62 | 6.50E-02 | 0.091 |
| | Age x Whole-body Tasks effect | 0.36 | 7.01E-01 | 0.010 |
| **G. Negativity Index** / **ANCOVA Age x Whole-body Distances** | Whole-body Distances effect | 0 | 9.68E-01 | 0.000 |
| | Age effect | 4.42 | 4.17E-02 | 0.099 |
| | Age x Distance effect | 2.85 | 9.90E-02 | 0.067 |

**Experiment 2**

| | Effects | F | p-value | Effect size (partial $\eta^2$) |
|---|---|---|---|---|
| **A. Effort perception** 2,5km/h / **ANOVA Age x Equilibrium conditions** | Equilibrium conditions | 34.5 | 8.60E-07 | 0.476 |
| | Age effect | 23.1 | 2.43E-05 | 0.379 |
| | Age x Equilibrium effect | 5.8 | 2.08E-02 | 0.132 |
| **B. Effort perception** 2,5km/h vs 4km/h / **ANOVA Age x Equilibrium conditions** | Equilibrium conditions | 32.1 | 1.64E-06 | 0.458 |
| | Age effect | 1.1 | 3.01E-01 | 0.028 |
| | Age x Equilibrium effect | 3.1 | 8.77E-02 | 0.075 |
| **C. Net Metabolic Power** 2,5km/h / **ANOVA Age x Equilibrium conditions** | Equilibrium conditions | 65.1 | 9.32E-10 | 0.631 |
| | Age effect | 43.1 | 1.00E-07 | 0.532 |
| | Age x Equilibrium effect | 14.1 | 5.86E-04 | 0.270 |
| **D. Net Metabolic Power** 2,5km/h vs 4km/h / **ANOVA Age x Equilibrium conditions** | Equilibrium conditions | 74 | 1.87E-10 | 0.661 |
| | Age effect | 0.39 | 5.37E-01 | 0.100 |
| | Age x Equilibrium effect | 2.7 | 1.06E-01 | 0.067 |
| **E. Step Length** 2,5km/h / **ANOVA Age x Equilibrium conditions** | Equilibrium conditions | 10.2 | 2.93E-03 | 0.215 |
| | Age effect | 9.2 | 4.30E-03 | 0.200 |
| | Age x Equilibrium effect | 1.2 | 2.80E-01 | 0.031 |
| **F. Step Length** 2,5km/h vs 4km/h / **ANOVA Age x Equilibrium conditions** | Equilibrium conditions | 5.59 | 2.34E-02 | 0.131 |
| | Age effect | 86.2 | 1.00E-06 | 0.699 |
| | Age x Equilibrium effect | 2.0 | 1.60E-01 | 0.051 |
| **G. Step Length Variability** 2,5km/h / **ANOVA Age x Equilibrium conditions** | Equilibrium conditions | 1 | 3.33E-01 | 0.025 |
| | Age effect | 5.9 | 1.98E-02 | 0.138 |
| | Age x Equilibrium effect | 0.2 | 6.77E-01 | 0.005 |
| **H. Step Length Variability** 2,5km/h vs 4km/h / **ANOVA Age x Equilibrium conditions** | Equilibrium conditions | 1.6 | 2.19E-01 | 0.042 |
| | Age effect | 10.9 | 2.21E-03 | 0.232 |
| | Age x Equilibrium effect | 0.0 | 8.29E-01 | 0.001 |
| **I. Step Frequency** 2,5km/h / **ANOVA Age x Equilibrium conditions** | Equilibrium conditions | 4.7 | 3.66E-02 | 0.113 |
| | Age effect | 1.4 | 2.44E-01 | 0.037 |
| | Age x Equilibrium effect | 4.2 | 4.86E-02 | 0.101 |
| **J. Step Frequency** 2,5km/h vs 4km/h / **ANOVA Age x Equilibrium conditions** | Equilibrium conditions | 11.6 | 1.66E-03 | 0.243 |
| | Age effect | 26.5 | 1.00E-06 | 0.424 |
| | Age x Equilibrium effect | 0.8 | 3.65E-01 | 0.023 |

**Supplementary Table 3.** Details of the statistical analyses presented in the manuscript for both experiment 1 and experiment 2.

**Experiment 1. A**. ANOVA on the movement duration: Age × type of tasks (Young/old × Arm/whole-body), **B**. ANCOVA analysis on the negativity index: Age × type of tasks (Young/old × Arm/whole-body) with movement duration as covariate, **C**. ANCOVA analysis on the negativity index: Age × whole-body tasks (Young/old × STS,BTS/WBR short/WBR long) with movement duration as covariate, **D**. ANCOVA analysis on the negativity duration for whole-body tasks: Age × whole-body tasks (Young/old × STS,BTS/WBR short/WBR long) with movement duration as covariate, **E**. ANCOVA analysis on the negativity amplitude for whole-body tasks: Age × whole-body tasks (Young/old × STS,BTS/WBR short/WBR long) with movement duration as covariate, and **F**. ANCOVA analysis on the negativity frequency for whole-body tasks: Age × whole-body tasks (Young/old × STS,BTS/WBR short/WBR long) with movement duration as covariate.

**Experiment 2.** ANOVA: Age × Equilibrium conditions (Young/old × Unconstrained walking/walking along a line) for **A.** effort perception with all participants walking at 2.5km/h, **B**. effort perception with older participants walking at 2.5km/h and younger participants walking at 4km/h, **C.** Net Metabolic Power with all participants walking at 2.5km/h, **D**. Net Metabolic Power with older participants walking at 2.5km/h and younger participants walking at 4km/h, **E**. Step Length with all participants walking at 2.5km/h, **F**. Step Length with older participants walking at 2.5km/h and younger participants walking at 4km/h, **G**. Step Length Variability with all participants walking at 2.5km/h, **H**. Step Length Variability with older participants walking at 2.5km/h and younger participants walking at 4km/h, **I**. Step Frequency with all participants walking at 2.5km/h, **J**. Step Frequency with older participants walking at 2.5km/h and younger participants walking at 4km/h.

The table gathers the F values, p-values, and effect sizes (partial $\eta^2$).

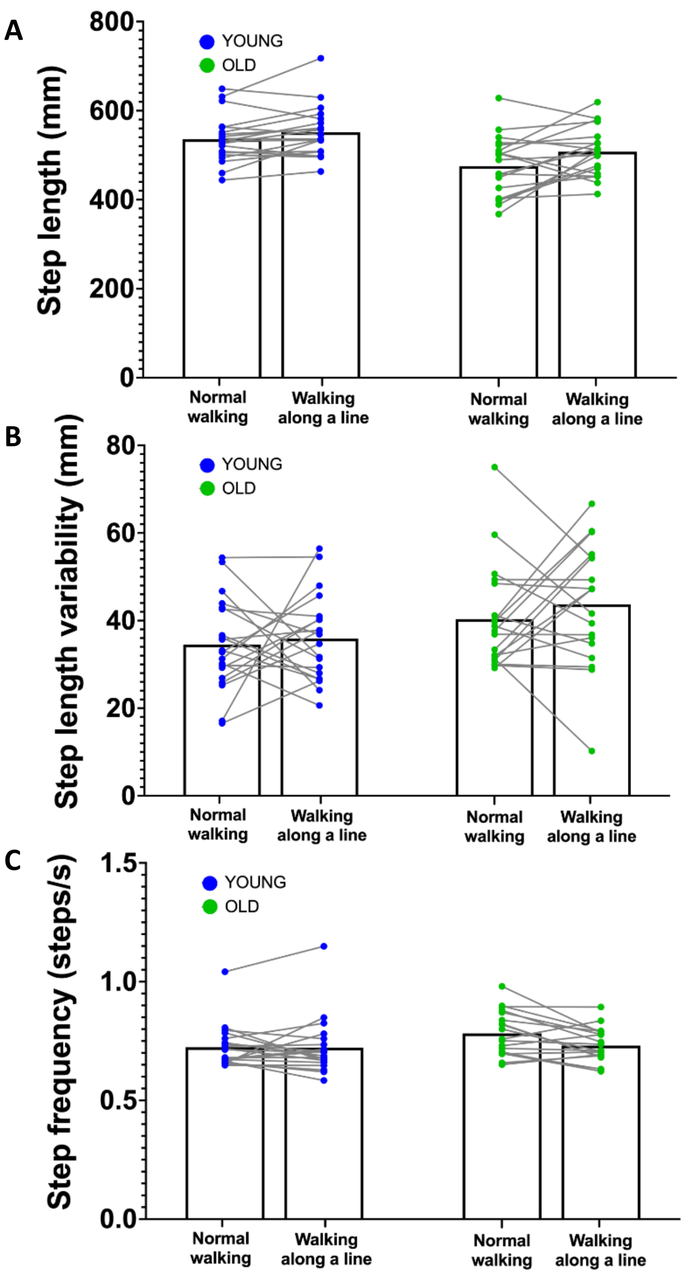


**Supplementary Figure 5. Step length, step length variability, and step frequency for participants walking at 2.5km/h.**

**A.** We found a main effect of Equilibrium Condition on **step length** ($F_{(1,37)}$=10.15, P=2.93E-3, $\eta^2$=0.215), indicating that participants adjusted their step length depending on the walking constraint. We also found an age effect ($F_{(1,37)}$=9.26, P=4.30E-3, $\eta^2$=0.200). However, there was no significant Age × Condition interaction ($F_{(1,37)}$=1.20, P=2.80E-1, $\eta^2$=0.031), and the same pattern was observed when comparing participants walking at different speeds (see Supplementary Table 3).

**B.** Older adults showed greater **step length variability** across conditions ($F_{(1,37)}$=5.9, P=1.98E-2, $\eta^2$=0.138), but no significant main effect of Condition ($F_{(1,37)}$=1.0, P=3.33E-1, $\eta^2$=0.025) or Age × Condition interaction was observed ($F_{(1,37)}$=0.2, P=6.77E-1, $\eta^2$=0.005). This result was consistent at both walking speeds.

**C. Step frequency** was significantly impacted by the equilibrium constraint ($F_{(1,37)}$=4.70, P=3.66E-2, $\eta^2$=0.113), with increased constraints leading to a decrease in frequency. This effect was more pronounced in older adults as revealed by the Age x Condition interaction ($F_{(1,37)}$=4.16, P=4.86E-2, $\eta^2$=0.101), suggesting greater sensitivity to balance demands. As expected, step frequency was higher in young participants walking at a faster speed.

Overall, older adults tended to take more steps and exhibited greater gait variability. The selected conditions appear appropriate, as they elicited differences between groups on two key measures.
